# β-arrestin-2 is an essential regulator of pancreatic β-cell function under physiological and pathophysiological conditions

**DOI:** 10.1038/ncomms14295

**Published:** 2017-02-01

**Authors:** Lu Zhu, Joana Almaça, Prasanna K. Dadi, Hao Hong, Wataru Sakamoto, Mario Rossi, Regina J. Lee, Nicholas C. Vierra, Huiyan Lu, Yinghong Cui, Sara M. McMillin, Nicole A. Perry, Vsevolod V. Gurevich, Amy Lee, Bryan Kuo, Richard D. Leapman, Franz M. Matschinsky, Nicolai M. Doliba, Nikhil M. Urs, Marc G. Caron, David A. Jacobson, Alejandro Caicedo, Jürgen Wess

**Affiliations:** 1 Molecular Signaling Section, Laboratory of Bioorganic Chemistry, National Institute of Diabetes and Digestive and Kidney Diseases, Bethesda, Maryland 20892, USA; 2 Division of Endocrinology, Diabetes and Metabolism, Department of Medicine, University of Miami Miller School of Medicine, Miami, Florida 33136, USA; 3 Department of Molecular Physiology and Biophysics, Vanderbilt University, Nashville, Tennessee 37232, USA; 4 Key Laboratory of Acupuncture and Medicine Research of Ministry of Education, Nanjing University of Chinese Medicine, Nanjing, Jiangsu 210023, China; 5 Mouse Transgenic Core Facility, National Institute of Diabetes and Digestive and Kidney Diseases, Bethesda, Maryland 20892, USA; 6 Department of Pharmacology, Vanderbilt University, Nashville, Tennessee 37232, USA; 7 Department of Molecular Physiology and Biophysics, University of Iowa, Iowa City, Iowa 52242, USA; 8 Laboratory of Bioengineering and Physical Science, National Institute of Biomedical Imaging and Bioengineering, Bethesda, Maryland 20892, USA; 9 Department of Biochemistry and Biophysics, University of Pennsylvania School of Medicine, Philadelphia, Pennslvania 19104, USA; 10 Department of Cell Biology, Duke University Medical Center, Durham, North Carolina 27710, USA

## Abstract

β-arrestins are critical signalling molecules that regulate many fundamental physiological functions including the maintenance of euglycemia and peripheral insulin sensitivity. Here we show that inactivation of the *β-arrestin-2* gene, *barr2*, in β-cells of adult mice greatly impairs insulin release and glucose tolerance in mice fed with a calorie-rich diet. Both glucose and KCl-induced insulin secretion and calcium responses were profoundly reduced in β-arrestin-2 (barr2) deficient β-cells. In human β-cells, *barr2* knockdown abolished glucose-induced insulin secretion. We also show that the presence of barr2 is essential for proper CAMKII function in β-cells. Importantly, overexpression of barr2 in β-cells greatly ameliorates the metabolic deficits displayed by mice consuming a high-fat diet. Thus, our data identify barr2 as an important regulator of β-cell function, which may serve as a new target to improve β-cell function.

## Abstract (web summary)

Beta-arrestins have key roles in development and metabolic functions as euglycaemic control and insulin sentitivity. Here Zhu *et al*. show that beta-arrestin-2 regulates insulin secretion and glucose tolerance in mice by promoting CAMKII functions in beta cells.

The two members of the β-arrestin family, β-arrestin-1 and -2 (barr1 and barr2; also known as arrestin-2 and arrestin-3, respectively) are widely expressed throughout the body^1^. Both β-arrestins regulate a wide array of important physiological functions^2^,^3^,^4^. It is well known that the β-arrestins bind to ligand-activated G-protein-coupled receptors (GPCRs) and that this process interferes with receptor/G protein coupling and promotes GPCR internalization via clathrin-coated pits^5^,^6^.

However, during the past decade, it has become increasingly clear that β-arrestins also represent signal transducers in their own right, primarily by acting as adaptor proteins for various signalling proteins and their effector pathways^3^,^7^,^8^,^9^,^10^. It is likely that these non-canonical β-arrestin functions can be exploited for the development of novel classes of clinically useful drugs, including β-arrestin-biased agonists^8^,^9^.

Studies with whole-body barr1 and barr2 knockout (KO) mice have shown that β-arrestins play important roles in several key metabolic functions including the maintenance of euglycemia and peripheral insulin sensitivity^4^,^11^,^12^. However, the metabolic phenotypes displayed by these mutant animals are often difficult to interpret, primarily for two reasons. First, β-arrestins are expressed in most tissues and cell types, making it difficult to determine which cellular pathways, in which particular tissues contribute to the observed metabolic deficits. Moreover, since the two β-arrestins regulate many important developmental functions^13^,^14^,^15^, it is also possible that the metabolic phenotypes displayed by adult whole-body barr1 and barr2 KO mice are modulated by compensatory developmental changes.

To circumvent these difficulties, we inactivated the *barr1* or *barr2* genes in a conditional fashion in specific, metabolically relevant cell types of adult mice. In the present study, we analysed a mouse strain in which we conditionally inactivated the *barr2* gene in β-cells of adult mice (β-barr2-KO mice). At present, very little is known about the potential role of barr2 in regulating β-cell function. Two recent studies reported contradictory results regarding the role of barr2 in modulating insulin secretion, probably due to problems associated with the use of whole-body barr2 KO mice (see above^16^,^17^). We hypothesized that detailed metabolic studies with β-barr2-KO mice should lead to unambiguous and novel insights into the role of β-cell barr2 in regulating β-cell function and whole-body glucose homoeostasis.

We found that β-barr2-KO mice show several striking metabolic deficits, including greatly impaired glucose-stimulated insulin secretion (GSIS) and Ca^2+^ entry into β-cells, and a pronounced reduction of glucose tolerance when β-barr2-KO mice consume a high-fat diet (HFD). We provide strong evidence that barr2 is required for the proper activation of CAMKII and that disruption of this pathway can fully account for the metabolic deficits observed with the β-barr2-KO mice. Moreover, knockdown of *barr2* expression virtually abolishes GSIS in human β-cells. Our findings may lead to the development of novel drugs aimed at modulating barr2 function in β-cells for therapeutic purposes.

## Results

### Conditional inactivation of barr2 in β-cells of adult mice

The two β-arrestins regulate many important developmental processes^14^,^15^,^18^. To avoid potential developmental changes due to barr2 deficiency, we used a conditional gene deletion strategy to selectively inactivate the *barr2* gene in β-cells of adult mice. Previous studies have shown that tamoxifen (TMX) induces Cre activity in *Pdx1-Cre-ER*^*TM*^ transgenic mice selectively in pancreatic β-cells^19^,^20^. We therefore crossed *Pdx1-Cre-ER*^*TM*^ mice (genetic background: C57BL/6) with homozygous floxed *barr2* mice, in which exon 2 was flanked by loxP sites (*fl/fl barr2* mice; genetic background: C57BL/6J; ref. 21). Subsequent matings led to the generation of *fl/fl barr2*-*Pdx1-Cre-ER*^*TM*^ mice and *fl/fl barr2* control littermates. Previous studies demonstrated that TMX-treated *Pdx1-Cre-ER*^*TM*^ mice do not show any changes in β-cell function, as compared with wild-type (wt) littermates^20^. For this reason, *fl/fl barr2* littermates served as control animals throughout this study. All animals used were maintained on a C57BL/6 background.

We injected *fl/fl barr2*-*Pdx1-Cre-ER*^*TM*^ mice and their control littermates (8-week-old males) for 6 consecutive days with TMX (1 mg i.p. per mouse per day) to induce Cre activity and *barr2* inactivation selectively in pancreatic β-cells^19^,^20^. Two weeks after the last TMX injection, we used quantitative real-time PCR (qRT-PCR) to determine *barr2* expression levels in different mouse tissues. As expected, *barr2* transcript levels were greatly reduced in pancreatic islets from TMX-treated *fl/fl barr2*-*Pdx1-Cre-ER*^*TM*^ mice, as compared with TMX-treated control littermates (*fl/fl barr2* mice; Supplementary Fig. 1a). The expression of islet barr2 protein was also dramatically reduced in the TMX-treated *fl/fl barr2*-*Pdx1-Cre-ER*^*TM*^ mice (Supplementary Fig. 1b). Most likely, the residual expression of *barr2* in the *fl/fl barr2*-*Pdx1-Cre-ER*^*TM*^ islets is due to *barr2* expression by islet cells that are non-β-cells (that is, α-cells). We also found that TMX-induced reduction of *barr2* expression was selective for islets/β-cells (Supplementary Fig. 1a). Importantly, deletion of the *barr2* gene in mouse islets/β-cells did not lead to significant compensatory changes in *barr1* transcript or protein levels in islets or other tissues (Supplementary Fig. 1b,c). For the sake of simplicity, we refer to the TMX-treated *fl/fl barr2*-*Pdx1-Cre-ER*^*TM*^ mice as ‘β-barr2-KO mice' below.

### Stimulated insulin release is impaired in β-barr2-KO islets

Immunohistochemical studies demonstrated that the lack of barr2 in β-cells had no detectable effect on overall islet/β-cell architecture (Supplementary Fig. 2). Similarly, β-cell barr2 deficiency had no significant effect on total pancreatic insulin (Supplementary Fig. 3) and islet insulin content (ng insulin per islet: β-barr2-KO, 142±9; control, 133±10; 26 independent islet preparations from six mice per group (8-week-old males)).

**Figure 1:**
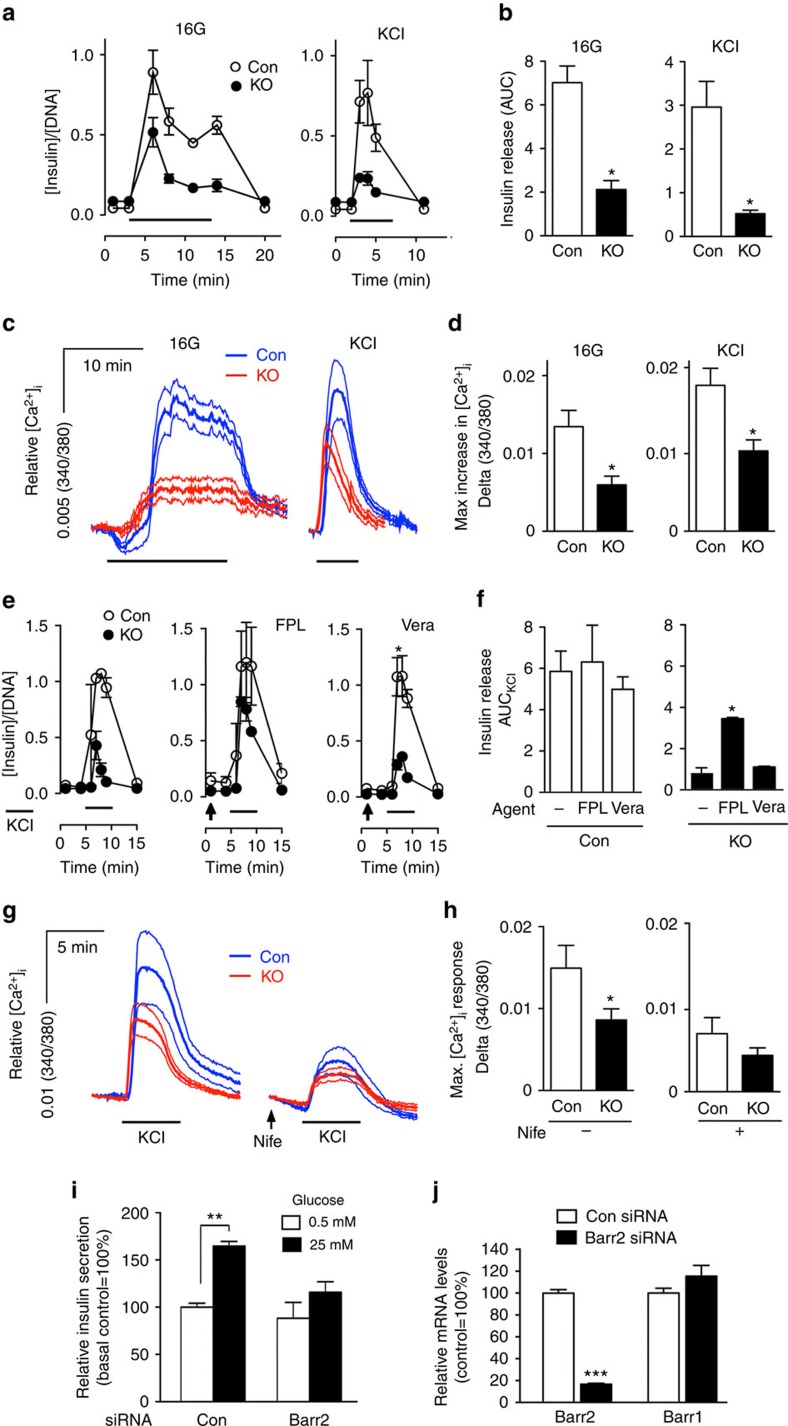
β-barr2-KO islets show reduced insulin and [Ca^2+^]_i_ responses and impaired function of LTCCs. (**a**) Insulin secretion studied with perifused pancreatic islets. Insulin secretion was studied using islets from control and β-barr2-KO mice stimulated with 16 mM glucose (16G) for 15 min or with KCl (25 mM) for 5 min, respectively. Thick horizontal bars indicate the 16G/KCl stimulation periods. All data are given as means±s.e.m. Insulin secretion was normalized to DNA content (*n*=3 perifusions per genotype; islets were isolated from six male mice per genotype). (**b**) Quantification of the data shown in **a**; *P<0.05 (Student's *t*-test). Data are given as means±s.e.m. (**c**) Increases in [Ca^2+^]_i_ studied with isolated pancreatic islets. Increases in [Ca^2+^]_i_ were recorded using control and β-barr2-KO islets following glucose (16G) or KCl (25 mM) stimulation, respectively. Thick horizontal bars indicate the 16G/KCl stimulation periods. Traces represent average responses (thick lines in the middle) and associated s.e.m. (thin lines above and below the thick lines) from 8 to 12 islets. Islets were isolated from six male mice per genotype. (**d**) Quantification of the data in **c** showing maximum [Ca^2+^]_i_ amplitudes after glucose or KCl stimulation. *P<0.05 (Student's *t*-test). (**e**) KCl-induced insulin secretion studied with perifused pancreatic islets under different experimental conditions. Insulin secretion from islets of control and β-barr2-KO mice was stimulated with KCl (25 mM; thick horizontal bars), either in the absence of other drugs (left panel), or in the presence of the selective LTCC activator FPL64176 (FPL, 10 μM; centre panel), and the selective Na^+^ channel opener veratridine (Vera, 10 μM; right panel). Arrows indicate when drugs were added to the medium. All data represent means±s.e.m. (*n*=3 perifusions per condition; islets were isolated from six male mice per genotype). Asterisks denote significant differences at time points 7–9 min. *P<0.05 (two-way repeated measures ANOVA followed by Bonferroni post tests). (**f**) Quantification of the data shown in **e**; *P<0.05. (**g**) KCl-induced increases in [Ca^2+^]_i_ studied with isolated pancreatic islets under different experimental conditions. Traces of [Ca^2+^]_i_ responses to KCl (25 mM) in the absence (left panel) or presence (right panel) of the selective LTCC blocker nifedipine (Nife, 10 μM; arrow). Traces represent average responses (thick lines in the middle) and associated s.e.m. (thin lines above and below the thick lines). Islets were isolated from six male mice per genotype (*n*=6–8 islets per genotype). (**h**) Quantification of the data in **c** showing maximum [Ca^2+^]_i_ amplitudes following KCl stimulation in the absence (left panel) or presence of nifedipine (Nife; right panel). Data represent means±s.e.m. (*P<0.05, Student's *t*-test). (**i**) *Barr2* knockdown greatly reduces GSIS in human β-cells. Human EndoC-βH1 cells were stimulated with glucose (25 mM) after treatment of cells with scrambled control siRNA (Con) or *barr2* siRNA (means±s.e.m., *n*=3; **P<0.01, as compared with the indicated control group, two-way ANOVA followed by Tukey's post-test). (**j**) Effective knockdown of *barr2* expression in human EndoC-βH1 cells following treatment with *barr2* siRNA (con=scrambled control siRNA). *barr2* and *barr1* mRNA levels were determined via real-time qRT-PCR and were normalized relative to the expression of *GAPDH*. Data are expressed as means±s.e.m. (*n*=3; ***P<0.001, Student's *t*-test). ANOVA, analysis of variance; AUC, area under the curve.

To assess the effects of barr2 deficiency on β-cell function, we performed islet perfusion experiments. We made the striking observation that glucose (16 mM)-induced insulin release was greatly reduced in the β-barr2-KO islets, as compared with control islets (Fig. 1a,b). Calcium imaging experiments demonstrated that glucose-induced increases in intracellular calcium levels ([Ca^2+^]_i_) were also severely blunted in the mutant islets (Fig. 1c,d).

Exposure of β-cells to high concentrations of glucose leads to enhanced glucose metabolism, closure of ATP-sensitive K^+^ channels (K_ATP_ channels), β-cell membrane depolarization, influx of Ca^2+^ through voltage-gated calcium channels, and an increase in [Ca^2+^]_i_, which in turn triggers the exocytosis of insulin-containing granules. To test the hypothesis that the functional impairments displayed by β-barr2-KO islets were caused by signalling deficits downstream of glucose-induced closure of K_ATP_ channels, we directly depolarized β-cell membranes by treating perifused islets with KCl (25 mM). Strikingly, KCl-induced insulin secretion and increases in [Ca^2+^]_i_ were reduced to a similar degree in β-barr2-KO islets, as compared with glucose-treated β-barr2-KO islets (Fig. 1a–d). This observation strongly suggests that barr2 facilitates insulin release through a pathway that is located downstream of glucose-induced inactivation of K_ATP_ channels.

### Barr2 promotes β-cell L-type Ca^2+^ channel function

Since the lack of β-cell barr2 led to greatly diminished glucose- and KCl-induced increases in [Ca^2+^]_i_, we hypothesized that Ca^2+^ influx was impaired in β-cell barr2-KO islets. GSIS is driven mainly by Ca^2+^ influx via L-type Ca^2+^ channels (LTCCs; ref. 22). To examine the potential role of LTCCs in the functional deficits displayed by barr2-KO islets, we treated islets with FPL64176, a selective activator of LTCCs. In this set of experiments, insulin secretion was triggered by direct membrane depolarization with KCl (25 mM). FPL64176 had no significant effect on KCl-stimulated insulin secretion in control islets (Fig. 1e,f). In contrast, FPL64176 greatly amplified the weak insulin response observed with KCl-stimulated β-barr2-KO islets (Fig. 1e,f). For control purposes, we carried out similar experiments with veratridine, a selective activator of Na^+^ channels. In contrast to FPL64176, veratridine had no significant effect on KCl-induced insulin release in control or β-barr2-KO islets (Fig. 1e,f).

As already shown in Fig. 1d, KCl-induced elevations in [Ca^2+^]_i_ were greatly reduced in barr2-deficient β-cells (Fig. 1g,h). Strikingly, in the presence of nifedipine (10 μM), a selective blocker of LTCCs, KCl-induced [Ca^2+^]_i_ responses were not significantly different in β-barr2-KO and control islets (Fig. 1g,h).

Taken together, the outcome of these pharmacological studies strongly suggests that the lack of barr2 interferes with the proper function of LTCCs in β-cells.

In mouse β-cells, the predominant LTCCs are Ca_V_1.2 and Ca_V_1.3 (refs 23, 24). qRT-PCR studies with RNA prepared from β-barr2-KO and control islets demonstrated that the expression levels of *Ca*_*V*_*1.2* and *Ca*_*V*_*1.3* (α-subunits) remained unaffected by the lack of barr2 (Supplementary Fig. 4, top row). This observation indicates that the impaired activity of LTCCs observed with β-barr2-KO islets is not due to altered LTCC expression levels. Supplementary Fig. 4 also shows that the expression levels of other key β-cell genes remained unaffected by the lack of barr2 in β-cells.

### *Barr2* knockdown greatly reduces GSIS in human β-cells

To confirm that barr2 is also a critical regulator of insulin release in human β-cells, we carried out insulin secretion studies with EndoC-βH1 cells, an immortalized human pancreatic β-cell line^25^. Treatment of EndoC-βH1 cells with *barr2* siRNA resulted in a ∼80% reduction in *barr2* expression while *barr1* transcript levels remained unaffected (Fig. 1j). Following treatment with 25 mM glucose, control EndoC-βH1 cells treated with scrambled control siRNA showed a significant increase in GSIS (Fig. 1i). In contrast, GSIS was virtually abolished in cells treated with *barr2* siRNA (Fig. 1i), indicating that barr2 is also essential for insulin release in human β-cells.

### EM analysis of β-cell dense core vesicles

Since glucose/KCl-stimulated insulin secretion was impaired in β-barr2-KO islets, we next examined whether the lack of barr2 affected the total number of β-cell dense core vesicles (DCVs) and the density of plasma membrane-docked DCVs. To obtain these parameters, we employed serial block-face scanning electron microscopy (SBF-SEM) using pancreatic islets prepared from control and β-barr2-KO mice (see Methods for details). This analysis demonstrated that the total number of β-cell DCVs remained unaffected by the absence of barr2 (Supplementary Fig. 5a–c). Similarly, control and barr2-deficient β-cells did not differ significantly from each other in the number of DCVs docked to the β-cell plasma membrane (Supplementary Figs 5d and 6). These data clearly indicate that barr2 deficiency does not affect the total number and distribution of β-cell DCVs.

### Lack of β-cell barr2 causes electrophysiological deficits

**Figure 2:**
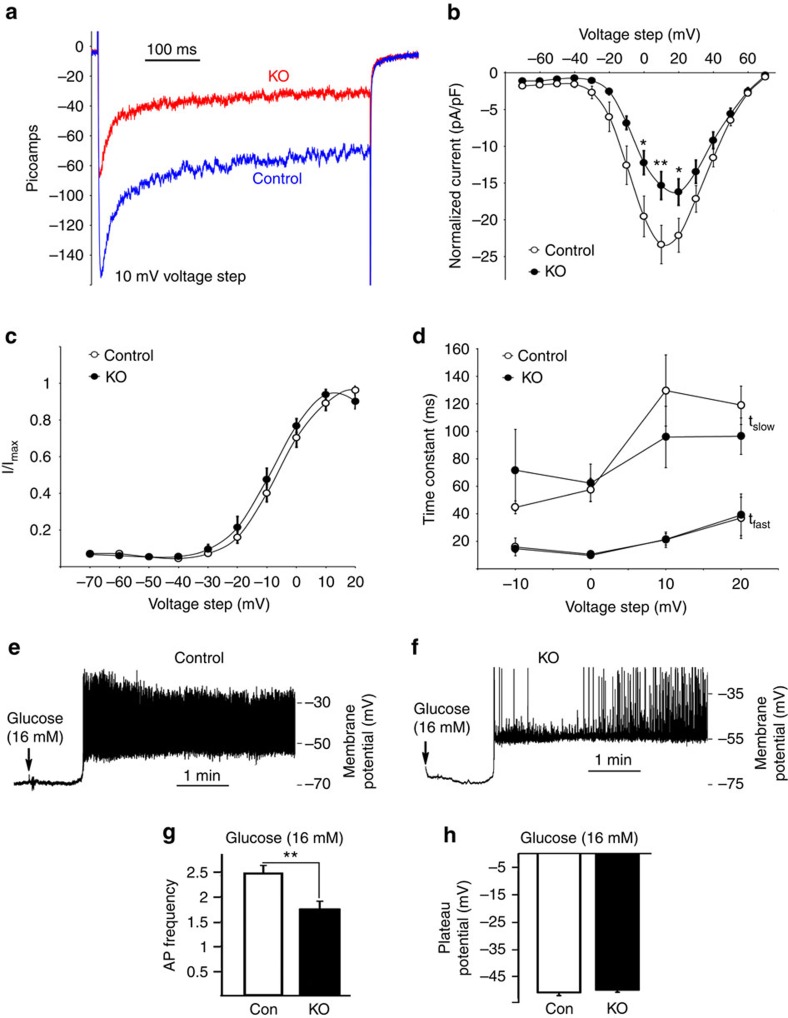
Lack of barr2 in β-cells causes a strong reduction in VDCC amplitude and AP firing frequency. (**a**) VDCC currents recorded from β-cells of control and β-barr2-KO mice in response to voltage steps of 10 mV from −70 to 70 mV. (**b**) Normalized average β-cell VDCC currents at the indicated voltage steps (control, *n*=15 islets; β-barr2-KO, *n*=13 islets). (**c**) Activation of β-cell VDCCs in response to the indicated voltage steps. (**f**) Time constants of VDCC inactivation in response to the indicated voltage steps (fast (*τ*_fast_) and slow (*τ*_slow_)). (**e**,**f**) Electrical activity of representative β-cells from a control (**e**) and a β-barr2-KO mouse (**f**) in response to 16 mM glucose. (**g**) β-Cell AP firing frequency recorded 2.5 min after glucose (16 mM) treatment (control, *n*=13 islets; β-barr2-KO, *n*=19 islets). (**h**) Plateau potential from where β-cell APs occurred 2.5 min after glucose (16 mM) treatment (control, *n*=13 islets; β-barr2-KO, *n*=19 islets). The data shown in **b**–**d**,**g** and **h** comprise experiments derived from five independent islet isolations using different sets of control and β-barr2-KO mice (adult males). Data represent means±s.e.m. (*P<0.05; **P<0.01; Student's *t*-test).

To directly study the role of barr2 in regulating the activity of β-cell voltage-dependent Ca^2+^ channels (VDCCs)/LTCCs, we carried out electrophysiological recordings studying β-cells from control and β-barr2-KO mice. Initially, we measured VDCC currents in response to 10 mV voltage steps from −70 to +70 mV. The β-cells were first held at −80 mV in low glucose (3 mM) for 3 min to limit Ca^2+^ influx and prevent Ca^2+^-induced changes in VDCC activity before the first recording. Interestingly, barr2-deficient β-cells showed significantly reduced VDCC currents in response to voltage steps between 0 and 20 mV when compared with control β-cells (Fig. 2a,b). Despite the decrease in VDCC currents, the lack of barr2 did not affect the kinetics of VDCC activation or inactivation (Fig. 2c,d). These data strongly support the concept that barr2 is required for efficient glucose-stimulated Ca^2+^ entry into β-cells by increasing the activity of VDCCs.

After glucose-dependent depolarization of the β-cell membrane, activation of VDCCs results in the upstroke of action potential (AP), which is the primary electrical signal of the β-cell^26^,^27^. To assess how changes in VDCC activity affect β-cell AP firing, we monitored the membrane potential of mouse β-cells in response to 16 mM glucose. The glucose-stimulated plateau potential from where APs occurred was similar for control and barr2-KO β-cells (−51.2±1.1 mV and −50.2±0.88 mV, respectively; Fig. 2h). In striking contrast, AP firing frequency measured 2.5 min after 16 mM glucose was greatly reduced in barr2-KO β-cells (1.73±0.17 Hz), as compared with control β-cells (2.47±0.14 Hz; Fig. 2e–g). These data clearly indicated that barr2 increases β-cell AP firing frequency by augmenting VDCC activity.

### Lack of barr2 has no effect on β-cell K^+^ channels

**Figure 3:**
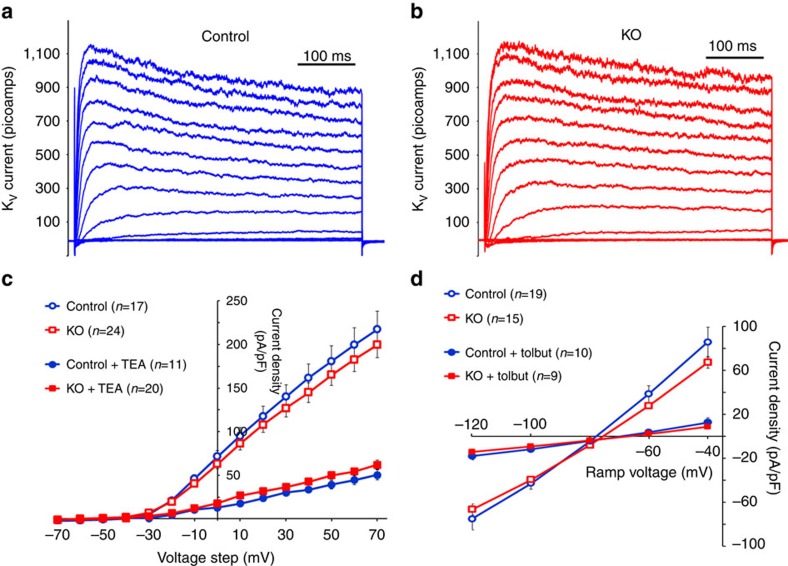
Barr2 deficiency does not affect β-cell K_v_ and K_ATP_ channel currents. (**a**) Representative K_v_ current traces from a control β-cell during 10 mV voltage steps from −70 to 70 mV. (**b**) Representative K_v_ current traces from a barr2-deficient (KO) β-cell during 10 mV voltage steps from −70 to 70 mV. (**c**) Summary of β-cell K_v_ currents recorded from control and KO β-cells at the indicated voltages and following inhibition with TEA (10 mM). Data are given as means±s.e.m. (**d**) K_ATP_ current recordings (induced by removal of intracellular ATP) from control and barr2-deficient (KO) β-cells at the indicated voltages and following inhibition with tolbutamide (Tolbut; 200 μM). Data are presented as means±s.e.m.

We next studied potential effects of barr2 deficiency on the activities of the two major β-cell K^+^ channels, the delayed rectifier voltage-gated K^+^ channel (K_V_) and the ATP-sensitive K^+^ channel (K_ATP_). K_v_ currents were recorded from control and barr2-deficient β-cells in response to 10 mV voltage steps from −70 to 70 mV (Fig. 3a,b). The resulting K_v_ currents were not significantly different between control and barr2-deficient β-cells. Moreover, K_v_ currents were inhibited by tetraethylammonium (TEA, 10 mM) to the same extent in the presence or absence of barr2 (Fig. 3c). We also recorded K_ATP_ currents from control and barr2 KO β-cells by removing intracellular ATP and recording the resulting K^+^ currents in response to a voltage ramp from −120 to −40 mV (Fig. 3d). This analysis showed that barr2 deficiency had no significant effect on the activity of K_ATP_ currents (Fig. 3d). Moreover, the lack of barr2 did not affect the ability of tolbutamide (200 μM) to inhibit K_ATP_ currents (Fig. 3d). Thus, the lack of barr2 in β-cells has no detectable effect on the activity of β-cell K_v_ or K_ATP_ channels.

### GPCRs promote GSIS in the absence of β-cell barr2

The activity of pancreatic β-cells is modulated by several GPCRs including the M_3_ muscarinic receptor^28^ and the GLP-1 receptor^29^. Since β-arrestins are well-known regulators of GPCR function, we studied whether M_3_ and GLP-1 receptor-mediated augmentation of GSIS was altered in β-barr2-KO islets. In the presence of a stimulatory concentration of glucose (16 mM), the muscarinic agonist, oxotremorine-M (Oxo-M; 10 μM), and GLP-1 (100 nM) amplified insulin secretion in a similar fashion in β-barr2-KO and control islets (Supplementary Fig. 7a–e), suggesting that barr2 deficiency does not interfere with the ability of these two GPCRs to augment GSIS.

Consistent with the insulin data, Ca^2+^ responses to Oxo-M and exendin-4, a GLP-1 analogue with increased hydrolytic stability, were significantly increased in the presence of a stimulatory concentration of glucose (16 mM; Supplementary Fig. 7f–j). Moreover, β-cell barr2 deficiency did not interfere with the ability of the two agonists to promote increases in [Ca^2+^]_i_ (Supplementary Fig. 7j). In fact, Oxo-M treatment of β-barr2-KO islets led to a significantly greater elevation of [Ca^2+^]_i_, as compared with Oxo-M-treated control islets (Supplementary Fig. 7j). The molecular mechanisms underlying this effect, which may involve impaired desensitization of the M_3_ muscarinic receptor, remain to be explored in future studies.

We also stimulated β-barr2-KO and control islets with Oxo-M (10 μM) in the absence of extracellular Ca^2+^. Under these conditions, muscarinic agonists, such as Oxo-M, stimulate increases in β-cell [Ca^2+^]_i_ via G_q_-dependent activation of IP_3_ receptors, triggering Ca^2+^ release from ER Ca^2+^ pools^30^. We found that the Oxo-M-induced increases in [Ca^2+^]_i_ were similar in β-barr2-KO and control islets (Supplementary Fig. 7k), suggesting that β-cell barr2 deficiency does not affect Ca^2+^ release from ER stores.

### β-barr2-KO mice show striking metabolic deficits *in vivo*

**Figure 4:**
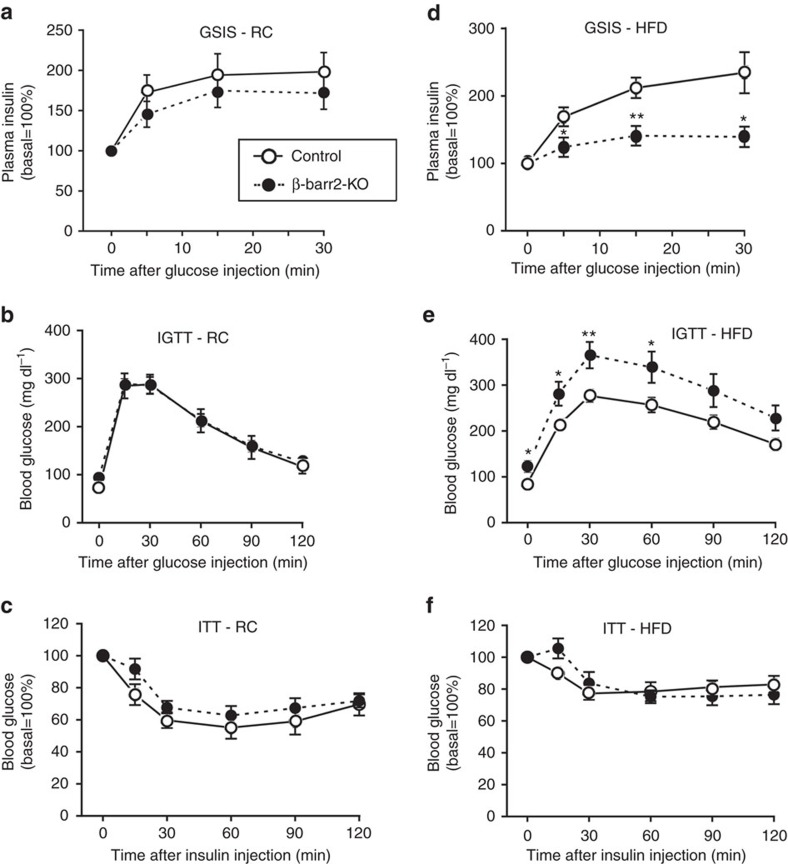
*In vivo* metabolic studies with mice lacking barr2 in pancreatic β-cells. (**a**–**c**) *In vivo* metabolic tests carried out with β-barr2-KO mice and control littermates maintained on RC. (**a**) Glucose-induced insulin secretion (GSIS; 2 g glucose per kg i.p.). (**b**) IGTT (2 g glucose per kg i.p.). (**d**) ITT (0.75 U insulin per kg i.p.). (**d**–**f**) β-barr2-KO mice and control littermates were maintained on a high-HFD and subjected to the same tests as in **a**–**c**. In **d** and **e**, the glucose dose was reduced to 1 g kg^−1^ (i.p.). In (**f**), the insulin dose was 1 U kg^−1^ (i.p.). Mice were maintained on the HFD for at least 8 weeks. All studies were carried out with male mice (mouse age: RC, 10–16 weeks; HFD, 16–22 weeks). Data are given as means±s.e.m. (8–12 mice per group). *P<0.05, **P<0.01, as compared with the corresponding control value (Student's *t*-test).

To investigate whether the functional impairments observed with β-barr2-KO islets *in vitro* were associated with metabolic deficits *in vivo*, we subjected β-barr2-KO and control mice to a series of *in vivo* metabolic studies. Interestingly, β-barr2-KO mice consuming regular chow (RC) showed only mild metabolic phenotypes (Fig. 4a–c). The mutant mice displayed a trend towards reduced GSIS (Fig. 4a). However, β-barr2-KO mice and control littermates showed a similar degree of glucose tolerance (i.p. glucose tolerance test (IGTT); Fig. 4b) and insulin sensitivity (0.75 U insulin per kg i.p.; Fig. 4c). Moreover, basal plasma insulin levels were not affected by β-barr2-deficiency (Supplementary Table 1).

In contrast, when β-barr2-KO mice were maintained on a calorie-rich, HFD, they showed pronounced metabolic impairments. Strikingly, GSIS was essentially abolished in HFD β-barr2-KO mice (Fig. 4d). Consistent with this finding, HFD β-barr2-KO mice displayed greatly impaired glucose tolerance and a significant increase in fasting blood glucose levels (Fig. 4e). In both groups of mice, the HFD induced a similar degree of reduced insulin sensitivity (Fig. 4f). β-Cell barr2 deficiency had no significant effect on basal plasma insulin levels (Supplementary Table 1).

Islet morphometric studies showed that β-cell mass was unaltered in HFD β-barr2-KO mice, as compared with HFD control littermates (Supplementary Fig. 8). Also, consumption of the HFD caused similar weight gain in control and barr2-KO mice (Supplementary Fig. 9). Collectively, these data strongly support the notion that the pronounced metabolic deficits observed with HFD β-barr2-KO mice are due to impaired insulin secretion, consistent with the *in vitro* insulin release studies.

### Barr2 is essential for β-cell function by regulating CAMKII

Our next goal was to identify the cellular pathway through which barr2 exerts its beneficial effects on β-cell function including insulin release. We recently found that conditional inhibition of CAMKII in β-cells leads to severe deficits in β-cell function, associated with impaired glucose homoeostasis *in vivo*^31^. Interestingly, the metabolic *in vitro* and *in vivo* phenotypes that we observed with this mouse model are strikingly similar to those displayed by the β-barr2-KO mutant mice. We therefore hypothesized that barr2 might be required for the proper activity of CAMKII in β-cells.

**Figure 5:**
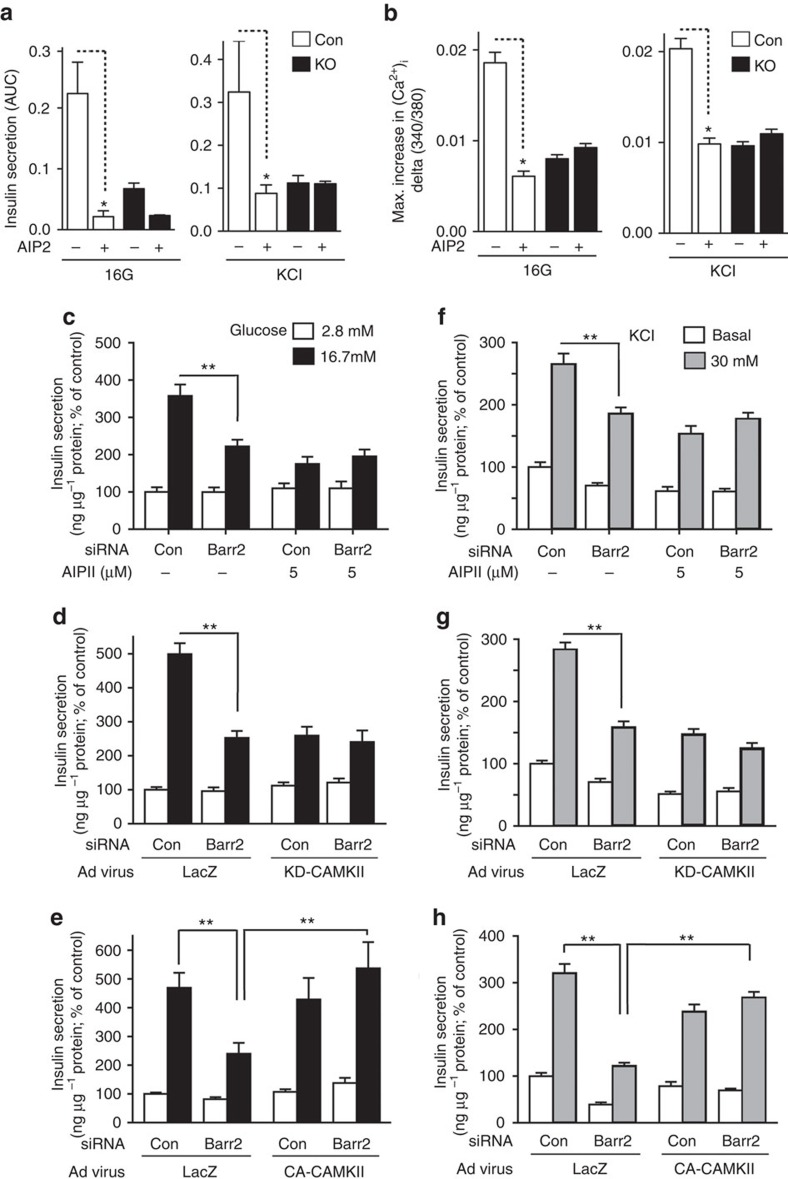
Role of CAMKII in mediating the stimulatory effects of barr2 in islets/β-cells. (**a**) Stimulation of insulin release by glucose (16G, left panel) and KCl (25 mM, right panel) from control and β-barr2-KO islets, in the absence or presence of a selective CAMKII inhibitor (AIP2, 5 μM). Islets were incubated for 30 min with AIP2 before glucose or KCl stimulation. AIP2 significantly inhibited insulin secretion in control islets but had no significant effect on insulin release from β-barr2-KO islets (*P<0.05, one-way ANOVA followed by Tukey's post-test; *n*=3 perifusions per condition; islets were isolated from six male mice per genotype; means±s.e.m.). (**b**) Maximum amplitudes of [Ca^2+^]_i_ responses (delta 340/380 nm) to 16G (left panel) and KCl (25 mM, right panel) in control and β-barr2-KO islets in the presence or absence of AIP2 (5 μM). Maximum [Ca^2+^]_i_ responses were defined as the difference between maximum and basal 340/380 nM values. AIP2 significantly inhibited 16G- and KCl-induced increases in [Ca^2+^]_i_ in control islets but had no significant effect on [Ca^2+^]_i_ responses in β-barr2-KO islets (*P<0.05, one-way ANOVA followed by Tukey's post-test; *n*=6 islets per condition; islets were isolated from six male mice per genotype; means±s.e.m.). (**c**–**e**) Glucose (16.7 mM)-stimulated insulin secretion by glucose (GSIS) in MIN6 cells (stimulation period: 1 h). Before insulin secretion studies, cells were treated with either scrambled control siRNA (Con) or *barr2* siRNA. (**c**) GSIS in the presence of a selective CAMKII inhibitor (AIP2, 5 μM). AIP2 significantly inhibited insulin secretion in control cells but had no effect on insulin release in *barr2* knockdown cells. (**d**) GSIS studied with cells infected with adenoviruses coding for KD-CAMKII (a dominant negative mutant of CAMKII) or LacZ (control). (**e**) GSIS studied with cells infected with adenoviruses coding for CA-CAMKII (a constitutively active version of CAMKII) or LacZ (control). (**f**–**h**) KCl-induced stimulation of insulin secretion in MIN6 cells (stimulation period: 1 h). Before insulin release studies, cells were treated with either scrambled control siRNA (Con) or *barr2* siRNA. Cells were exposed to AIP2 or different adenoviruses, as indicated in **a**–**c**. Data are given as means±s.e.m. from three independent experiments carried out in hexuplicate. *P<0.05, **P<0.01, as compared with the indicated control group (two-way ANOVA followed by Student's *t*-test). ANOVA, analysis of variance; AUC, area under the curve.

To test this hypothesis, we incubated β-barr2-KO and control islets with AIP2 (autocamtide-2 related inhibitory peptide II), a cell-permeable, selective peptide inhibitor of CAMKII (refs 31, 32, 33). We found that AIP2 (5 μM) treatment of control islets greatly reduced glucose- and KCl-dependent increases in insulin secretion and [Ca^2+^_i_] (Fig. 5a,b). Notably, these impairments were similar in magnitude to those observed with β-barr2-KO islets that had not been exposed to AIP2 (Fig. 5a,b). In contrast to the control islets, β-barr2-KO islets showed little or no sensitivity to AIP2 treatment (Fig. 5a,b).

We obtained very similar results when we studied the effect of AIP2 on GSIS and KCl-induced insulin secretion in cultured mouse β-cells (MIN6 cells) treated with either scrambled control siRNA or *barr2* siRNA (Fig. 5c,f). qRT-PCR experiments showed that *barr2* siRNA-mediated knockdown of *barr2* expression in MIN6 cells did not lead to compensatory changes in *barr1* expression levels (Supplementary Fig. 10). Besides using a peptide inhibitor (AIP2), we also disrupted CAMKII function by infecting MIN6 cells with an adenovirus coding for a dominant negative version of CAMKII (KD-CAMKII; ref. 34). A pharmacologically inert adenovirus coding for lacZ was used for control purposes. This alternative strategy to inhibit CAMKII function resulted in a pattern of insulin responses that was very similar to that seen with AIP2-treated MIN6 cells or pancreatic islets (Fig. 5d,g).

The CAMKII inhibition data strongly suggested that barr2 is a component of a β-cell signalling pathway that is required for CAMKII activation. To examine whether barr2 acts upstream or downstream of CAMKII, we infected MIN6 cells treated with either scrambled control siRNA or *barr2* siRNA with an adenovirus coding for a constitutively active version of CAMKII (CA-CAMKII; ref. 34). Expression of CA-CAMKII in MIN6 cells treated with control siRNA had no significant effect on glucose (16.7 mM)- or KCl (30 mM)-induced insulin secretion (Fig. 5e,h). Remarkably, expression of CA-CAMKII in cells treated with *barr2* siRNA completely rescued the pronounced deficits in glucose- and KCl-induced insulin secretion caused by *barr2* knockdown (Fig. 5e,h). In contrast to AIP2, a membrane-permeable control peptide (*Drosophila* antennapedia homeodomain leader peptide) had no significant effect on glucose- and KCl-stimulated insulin secretion in MIN6 cells (note that this sequence is part of the AIP2 peptide) (Supplementary Fig. 11).

**Figure 6:**
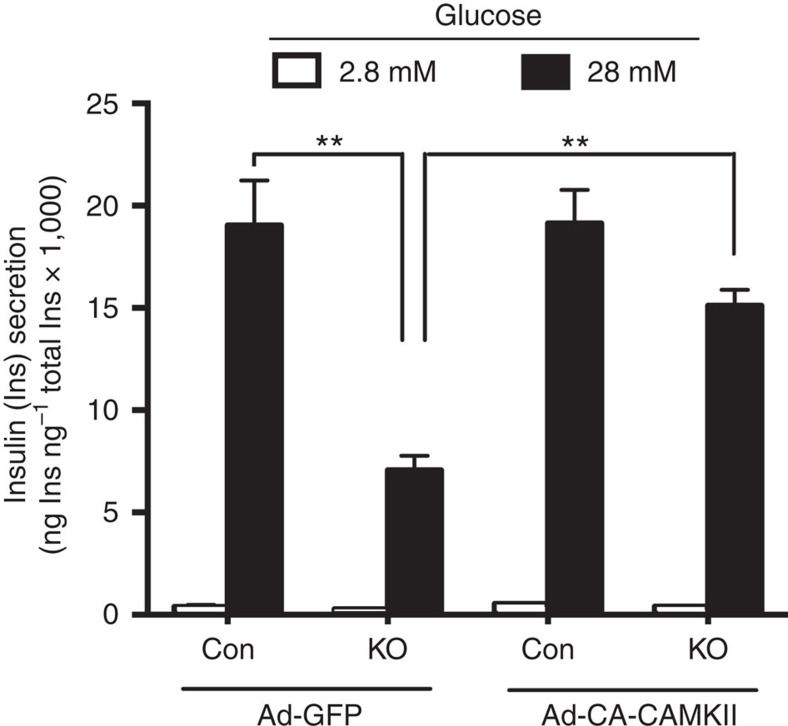
Deficits in GSIS in β-barr2 KO islets are rescued by a constitutively active CAMKII mutant (CA-CAMKII). Islets prepared from β-barr2 KO mice or control littermates were infected with adenoviruses coding for CA-CAMKII or GFP (control). After pre-incubation of islets with insulin secretion buffer for 30 min, islets were incubated in low or high-glucose buffer (2.8 or 28 mM glucose, respectively) for 1 h. Data are given as means±s.e.m. (*n*=4–8). **P<0.01, as compared with the indicated control group (two-way ANOVA followed by Student's *t*-test). ANOVA, analysis of variance.

We next examined whether the deficits in GSIS observed with β-barr2 KO islets could also be rescued by a constitutively active version of CAMKII. Specifically, we infected β-barr2 KO and control islets with adenoviruses coding for CA-CAMKII or GFP (control; Fig. 6). After a 30 min pre-incubation, the islets were incubated in low- or high-glucose buffer (2.8 or 28 mM glucose, respectively) for 1 h. We found that treatment of β-barr2 KO islets with the CA-CAMKII virus efficiently rescued the impairment in GSIS caused by barr2 deficiency (Fig. 6).

Taken together, these findings strongly suggest that barr2 acts upstream of CAMKII in pancreatic β-cells.

### Barr2 deficiency prevents synapsin I phosphorylation

**Figure 7:**
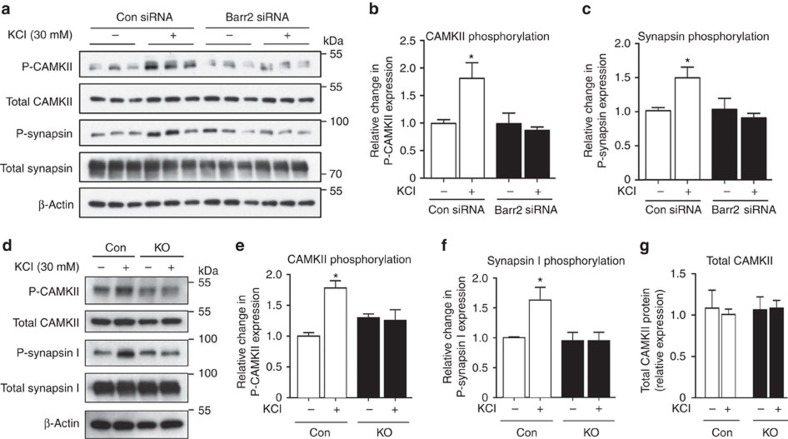
CAMKII and synapsin I phosphorylation and barr2/CAMKII co-immunoprecipitation/co-localization studies. (**a**–**f**) Western blotting studies. (**a**–**c**) KCl (30 mM) stimulation of MIN6 cells treated with scrambled control siRNA (Con) promotes the phosphorylation of CAMKII (**a**,**b**) and synapsin I (**a**,**c**). These responses are abolished after treatment of MIN6 cells with *barr2* siRNA. (**b**,**c**) Quantification of CAMKII and synapsin I phosphorylation data derived from immunoblotting experiments normalized by total CAMKII or total synapsin I expression, respectively. (**d**–**f**) Data represent means±s.e.m. of three independent experiments carried out in triplicate. *P<0.05, **P<0.01, as compared with the non-stimulated control group (one-way ANOVA followed by Tukey's post-test). (**d**–**f**) Western blotting experiments similar to those shown in **a**–**c** were carried out with pancreatic islets prepared from barr2-KO mice (KO) and control littermates (12-week-old males). (**g**) Total CAMKII expression levels in control and β-barr2 KO islets determined in immunoblotting studies using a pan-CAMKII antibody. Protein levels are expressed relative to the expression of β-actin. Data represent means±s.e.m. of three or four independent experiments (**b**,**c**,**e**–**g**). *P<0.05, as compared with the non-treated control group (one-way ANOVA followed by Tukey's post-test). ANOVA, analysis of variance.

CAMKII is known to stimulate insulin secretion via phosphorylation of various signalling proteins involved in insulin exocytosis, including synapsin I (refs 31, 35, 36). CAMKII activation is also associated with its auto-phosphorylation at Thr-286 (refs 37, 38). Consistent with these findings, western blotting studies demonstrated that KCl (30 mM) treatment of MIN6 cells increased both CAMKII auto-phosphorylation and synapsin I phosphorylation (Fig. 7a–c). Strikingly, these phosphorylation events were completely abolished in barr2-deficient cells (Fig. 7a–c). We obtained similar results when we carried out CAMKII and synapsin I phosphorylation studies with control and β-barr2-KO islets (Fig. 7d–f). Importantly, total CAMKII levels were similar in control and β-barr2 KO islets (Fig. 7g).

### Stimulated CAMKII activity is reduced in β-barr2 KO islets

We also performed CAMKII activity assays with control and β-barr2 KO islets. These experiments were carried out at two different concentrations of glucose (2.8 and 28 mM, respectively), either in the presence or absence of Ca^2+^/calmodulin. The lack of barr2 had no significant effect on total CAMKII activity measured in the presence of Ca^2+^/calmodulin (Supplementary Fig. 12a), consistent with the observation that β-cell barr2 deficiency did not affect total CAMKII expression levels (Fig. 7g). However, in the presence of a stimulatory concentration of glucose (28 mM), β-barr2 KO islets displayed a significant reduction in autonomous (Ca^2+^/calmodulin-independent) CAMKII activity (Supplementary Fig. 12b), in good agreement with the results of the CAMKII auto-phosphorylation studies (Fig. 7e).

### Detection of barr2/CAMKII complexes in mouse islets

**Figure 8:**
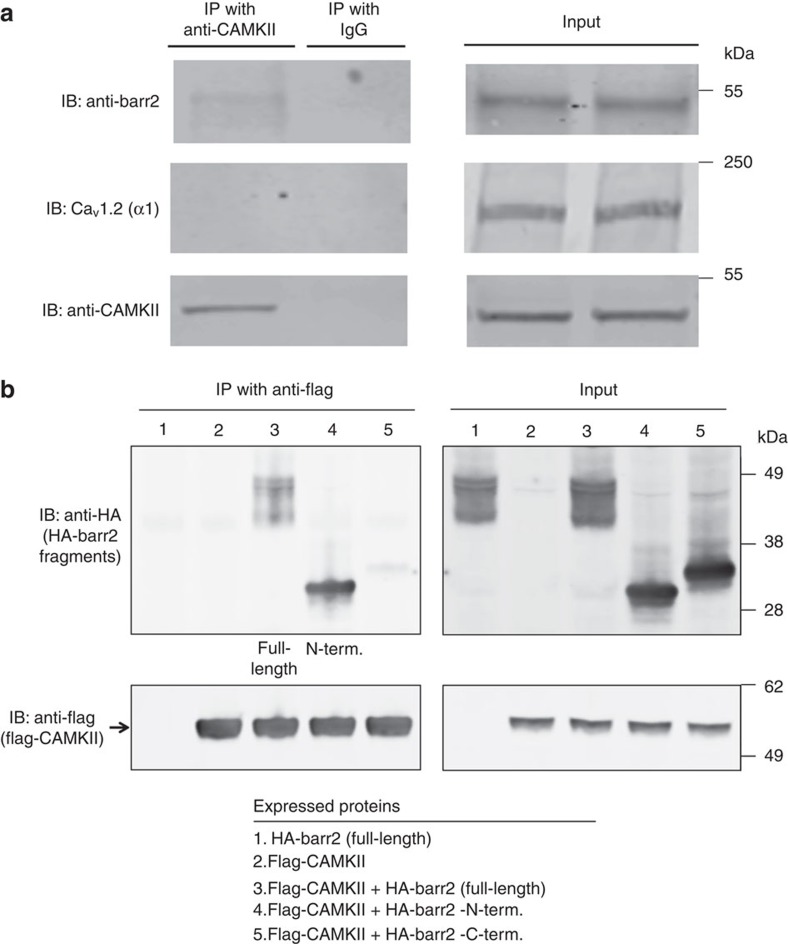
Co-immunoprecipitation of barr2/CAMKII complexes. (**a**) Studies with wt mouse pancreatic islets. Lysates prepared from wt mouse pancreatic islets were subjected to immunoprecipitation with either an anti-CaMKIIδ antibody or goat IgG (negative control). Immunoprecipitated proteins were then probed with anti-barr2, anti-CaMKII (pan), and anti-Ca_V_1.2 (α1-subunit^69^) antibodies. Note that barr-2, but not Ca_V_1.2, could be co-immunoprecipitated with CAMKII. (**b**) Co-immunoprecipitation studies with transfected HEK293T cells. HEK293T cells were transfected with the indicated plasmids. All barr2 constructs carried an N-terminal HA epitope tag (HA-barr2-N-term: barr2 residues 1–181; HA-barr2-C-term: barr2 residues 180–408). In addition, cells were infected with an adenovirus coding for flag-CaMKIIδ (lanes 2–5) or an empty control virus (lane 1). Flag-CaMKIIδ was immunoprecipitated from cell lysates with an anti-flag antibody, followed by immunoblotting studies to detect co-immunoprecipitated barr2/barr2 fragments. This analysis indicated that the barr2-N-domain (similar to full-length barr2), but not the barr2-C-domain, was clearly detectable in the immunoprecipitates. The blots shown are representative of two or three independent experiments.

We next used a co-immunoprecipitation strategy to explore the possibility that barr2 can form a complex with CAMKII in native mouse β-cells (pancreatic islets). Specifically, we subjected lysates prepared from wt mouse pancreatic islets to immunoprecipitation with either an anti-CaMKIIδ antibody or goat IgG (negative control). Immunoprecipitated proteins were then probed via western blotting with an anti-barr2 antibody. Using this approach, we detected barr2 protein as a ∼50 kDa species in the immunoprecipitates (Fig. 8a). As expected, control IgG immunoprecipitates did not yield any detectable immunoreactive bands (barr2 or CaMKIIδ), indicative of the selectivity of the CaMKIIδ antibody used (Fig. 8a). These findings strongly support the existence of barr2/CAMKII complexes in native mouse islets (β-cells).

In mouse pancreatic β-cells, the Ca_V_1.2 channel is one of the predominant LTCCs^23^,^24^. To examine whether barr2 has access to Ca_V_1.2 in native β-cells, we subjected lysates prepared from wt mouse pancreatic islets to the same immunoprecipitation strategy described above. By using anti-Ca_V_1.2 (α_1_-subunit) and anti-barr2 antibodies, we were unable to demonstrate the existence of barr2/Ca_V_1.2 complexes (Fig. 8a). This observation indicates that barr2 does not have access to LTCCs.

### Role of the N-terminal barr2 domain in CAMKII complexes

β-arrestins consist of two major regions^1^, the N- and C-terminal domains (barr2-N-domain: residues 1–181; barr2-C-domain: 180–408). To explore which of these two domains is involved in the formation of protein complexes containing barr2 and CAMKII, we carried out additional co-immunoprecipitation studies using co-transfected HEK293T cells. Cells were transfected with plasmid DNAs coding for full-length barr2, the barr2-N-domain, or the barr2 C-domain (all constructs carried an N-terminal HA tag). Subsequently, cells were infected with an adenovirus coding for flag-CAMKII or a control virus. Following cell lysis, flag-CAMKII was immunoprecipitated with an anti-flag antibody. Western blotting studies showed that the barr2-N-domain, but not the barr2-C-domain, was clearly detectable in the immunoprecipitates (Fig. 8b). This finding suggests that the N-domain of barr2 is critically involved in the formation of protein complexes containing barr2 and CAMKII.

### Purified barr2 does not interact with purified CAMKII

To examine whether barr2 was able to bind to CAMKII directly, we studied the ability of purified barr2 fused to the C-terminus of maltose-binding protein (MBP-barr2; ref. 39) to interact with purified CAMKII (CAMKIIδ). Pull-down assays failed to demonstrate a direct interaction between MBP-barr2 and CAMKII (Supplementary Fig. 13a). In contrast, as reported previously^39^, purified MBP-barr2 was able to bind to purified JNK3 (JNK3α2, positive control; Supplementary Fig. 13b).

### *In vivo* studies with mice overexpressing barr2 in β-cells

Since the lack of barr2 in β-cells led to impaired glucose homoeostasis (Fig. 4), we hypothesized that enhanced signalling via barr2 in β-cells might promote GSIS and improve glucose tolerance. To test this hypothesis, we generated transgenic mice which selectively overexpressed an HA-tagged version of barr2 in pancreatic β-cells (for details, see Methods; also see Supplementary Fig. 14). In the following, we refer to these mice as *RIPII-barr2* Tg mice.

**Figure 9:**
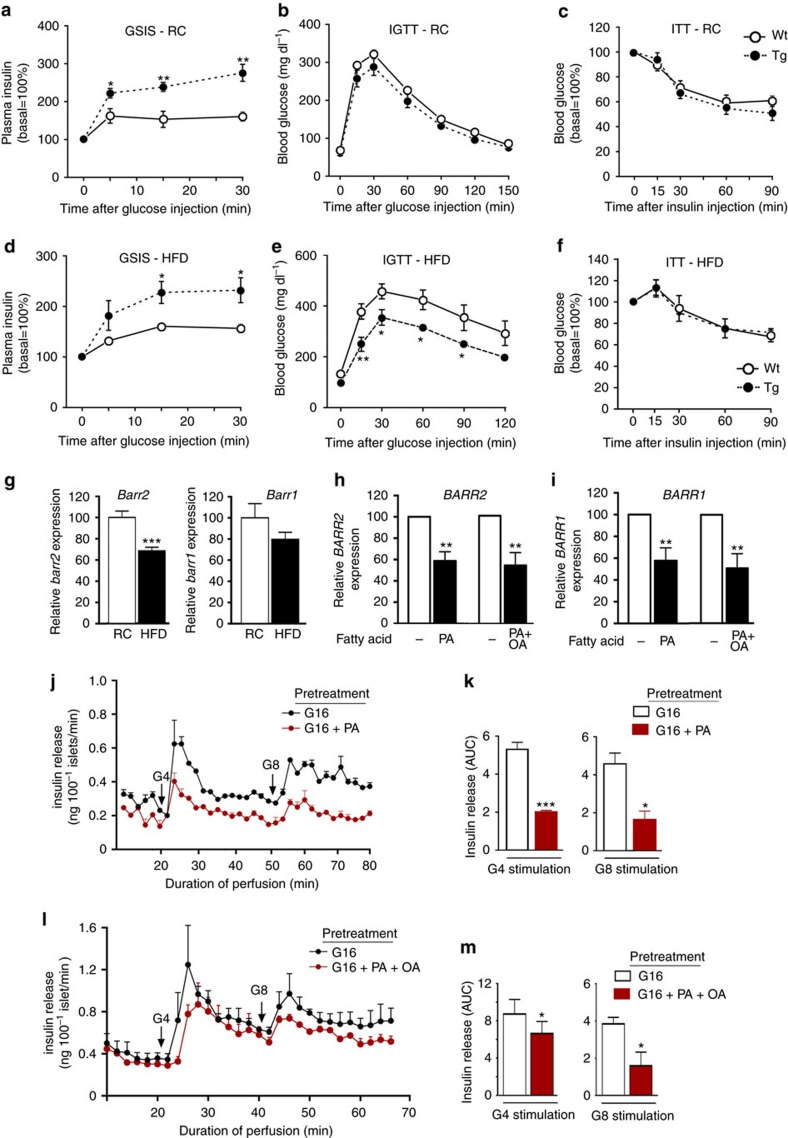
*In vivo* studies with transgenic mice overexpressing barr2 in pancreatic β-cells. (**a**–**c**) *In vivo* metabolic tests carried out with *RIPII-barr2* Tg mice (Tg) and wild-type (wt) littermates maintained on RC. (**a**) Glucose-induced insulin secretion (GSIS; 2 g glucose per kg i.p.). (**b**) IGTT (2 g glucose per kg i.p.). (**c**) ITT (0.75 U insulin per kg i.p.). (**d**–**f**) *RIPII-barr2* Tg mice (Tg) and wt littermates were maintained on a HFD and subjected to the same tests as in **a**–**c**. In **d** and **e**, the glucose dose was reduced to 1 g kg^−1^ (i.p.). In **f**, the insulin dose was 1 U kg^−1^ (i.p.). Mice were maintained on the HFD for at least 8 weeks. All studies were carried out with male mice (mouse age: RC, 8–12 weeks; HFD, 16–20 weeks). Data are given as means±s.e.m. (6–9 mice per group); *P<0.05, **P<0.01, as compared with the corresponding wt value (Student's *t*-test). (**g**) *Barr2* expression is reduced in islets of mice maintained on a HFD. Total RNA was prepared from pancreatic islets of wt mice (16-week-old male C57BL/6NTac mice; eight mice per group). Mice were maintained on either RC or on a HFD (for 8 weeks). Gene expression levels were determined via real-time qRT-PCR. Transcript levels were normalized relative to the expression of β*-actin* (for primer sequences, see Methods). Data represent means±s.e.m. (***P<0.001; Student's *t*-test). (**h**,**i**) Reduced β-arrestin expression in human islets cultured in glucose-rich medium (16 mM) in the presence of 0.5 mM palmitic acid (PA) or a 2:1 mixture of PA and oleic acid (PA+OA; total fatty acid concentration: 0.5 mM) for 3 days. Relative *BARR2* (**h**) and *BARR1* (**i**) expression levels were determined by qRT-PCR (internal control gene: β*-ACTIN)*. *BARR2* and *BARR1* expression levels determined with islets that had not been exposed to PA or PA+OA were set equal to 100 in each individual experiment. Data are given as means±s.e.m. (*n*=3 per group; islets were prepared from six different donors). **P<0.01, Student's *t*-test. (**j**,**l**) Impaired insulin secretion in human islets cultured in glucose-rich medium (16 mM) in the presence of 0.5 mM PA or a 2:1 mixture of PA and OA (total fatty acid concentration: 0.5 mM) for 3 days. Insulin release in response to 4 and 8 mM glucose (G4 and G8, respectively) was monitored using an islet perifusion system. Note that isolated human islets are far more sensitive to glucose than mouse or rat islets explaining why both G4 and G8 strongly promote insulin release in control islets. Data are given means±s.e.m. of three independent perifusion experiments. (**k**,**m**) Quantification of the data shown in (**j**,**l**). Insulin release following stimulation wth G4 or G8 is expressed as AUC. Data represent means±s.e.m. of three independent perifusion experiments (*P<0.05, ***P<0.001, Student's *t*-test). AUC, area under the curve.

We subjected *RIPII-barr2* Tg mice and their wt littermates to the same *in vivo* metabolic tests as β-barr2-KO mice. In general, the phenotypes displayed by the *RIPII-barr2* Tg mice were opposite to those observed with the β-barr2-KO mice (Fig. 9).

When mice were maintained on RC, GSIS was greatly enhanced in *RIPII-barr2* Tg mice, as compared with their wt littermates (Fig. 9a). The transgenic mice also showed a trend towards improved glucose tolerance (Fig. 9b). In an insulin tolerance test (ITT), *RIPII-barr2* Tg mice and wt littermates showed a similar degree of insulin sensitivity (Fig. 9c). Moreover, as compared with wt littermates, fed blood glucose levels were significantly lower in the transgenic mice consuming RC (Supplementary Table 1).

When maintained on a calorie-rich HFD*, RIPII-barr2* Tg mice and wt littermates showed a similar degree of weight gain (Supplementary Fig. 15). Moreover, insulin sensitivity remained unaffected by the presence of the *RIPII-barr2* transgene (ITT; Fig. 9f). Strikingly, however, the transgenic mice displayed both a pronounced increase in GSIS (Fig. 9d) and greatly improved glucose tolerance (Fig. 9e). Fed and fasting blood glucose levels were significantly lower in the HFD transgenic mice, as compared with the corresponding wt littermates (Supplementary Table 1). Thus overexpression of barr2 in β-cells greatly ameliorated the key metabolic deficits associated with the consumption of a HFD.

### Decreased *barr2* expression in islets from HFD mice

To examine whether the consumption of a HFD affects *barr2* expression in pancreatic islets, we carried out qRT-PCR studies using RNA prepared from islets of wt mice (16-week-old male C57BL/6NTac mice) maintained on RC or a HFD. We found that HFD mice showed a significant reduction (by ∼30%) of islet *barr2* expression (Fig. 9g). We also noted a trend towards reduced *barr1* expression levels in HFD mice (Fig. 9g).

### Glucolipotoxicity affects *BARR2/1* levels in human islets

Elevated blood glucose and fatty acid levels are known to have deleterious effects on β-cell function including GSIS^40^. This phenomenon, referred to as ‘glucolipotoxicity', is predicted to play an important role in the pathogenesis of type 2 diabetes (T2D)^40^. To mimic this process in human pancreatic islets *in vitro*, we exposed human islets for three days to high concentrations of glucose (16.7 mM) and palmitic acid (0.5 mM). Islet perifusion studies showed that GSIS was significantly reduced in human islets cultured in the presence of palmitic acid, as compared with islets cultured in its absence (Fig. 9j,k).

To explore whether glucolipotoxic conditions affected the expression levels of β-arrestins in human islets, we carried out qRT-PCR experiments using total RNA prepared from human islets that had been cultured for 3 days in glucose (16.7 mM)-rich medium either in the presence or absence of palmitic acid (0.5 mM). This analysis showed that both *BARR2* and *BARR1* expression were significantly reduced (by ∼30–40%) under glucolipotoxic conditions (Fig. 9h,i). We obtained very similar results when we exposed human islets to a 2:1 mixture of palmitic and oleic acid (total fatty acid concentration: 0.5 mM) (Fig. 9h,i,l,m).

## Discussion

Barr2, similar to barr1, can act as a scaffold to coordinate the activity of many important signalling proteins^3^,^7^,^41^. Here we report the novel finding that barr2 expressed by pancreatic β-cells is required for proper β-cell function, both in mouse and human β-cells. We provide strong evidence that the presence of β-cell barr2 is critical for the proper function of CAMKII, a multi-functional Ser/Thr protein kinase. This novel, non-canonical activity of barr2 does not seem to require barr2 interactions with β-cell GPCRs.

Several lines of evidence suggest that the *in vitro* and *in vivo* phenotypes displayed by the β-barr2-KO mice are caused by impaired CAMKII function. First, the biochemical, electrophysiological, and metabolic deficits displayed by the β-barr2-KO mice closely mimic those described for a mutant mouse strain expressing a dominant negative version of CAMKII selectively in β-cells^31^. These deficits also include impaired Ca^2+^ influx through LTCCs/VDCCs (Figs 1 and 2), suggesting that the deficits in Ca^2+^ influx caused by β-cell barr2 deficiency are most likely a consequence of impaired CAMKII activity. Consistent with this observation, co-immunoprecipitation studies using lysates from wt mouse islets demonstrated that barr2 forms a complex with CAMKII, but not with LTCCs (Fig. 8a). In addition, we found that expression of a constitutively active version of CAMKII in cultured β-cells (Fig. 5e,h) or wt mouse islets (Fig. 6) rescued the deficits in insulin secretion caused by barr2 deficiency. CAMKII also promotes insulin secretion via phosphorylation of various signalling proteins involved in insulin exocytosis, including synapsin I (refs 31, 35, 36). CAMKII activation is dependent on its auto-phosphorylation at Thr-286 (refs 37, 38). Strikingly, we found that both synapsin I phosphorylation and CAMKII auto-phosphorylation were abolished in barr2-deficient β-cells (Fig. 7a–f).

In this context, it should be mentioned that Xiao *et al*.^42^ demonstrated the formation of β-arrestin/CAMKII complexes following angiotensin II type 1a receptor activation in cultured cells. Moreover, Mangmool *et al*.^43^ reported that β-arrestin-CAMKII-Epac1 complexes are functionally important in the mouse heart.

Taken together, these observations strongly support the concept that barr2 is required for the proper function of CAMKII in pancreatic β-cells and that impaired CAMKII activity can fully account for the deficits displayed by the β-barr2-KO mutant mice. Our findings, in combination with previous studies^42^,^43^, are consistent with a model in which β-cell barr2, as part of a complex that includes CAMKII and probably other proteins, functions as a critical positive regulator of CAMKII function.

Hudmon *et al*.^44^ demonstrated that the pore-forming α subunit of LTCCs (Ca_v_α1.2) is a CAMKII substrate and that tethering of CAMKII to LTCCs facilitates Ca^2+^ influx. It is therefore possible that a similar mechanism is operative in pancreatic β-cells. However, this notion remains to be tested experimentally.

Interestingly, while islets prepared from β-barr2-KO mice showed pronounced deficits in calcium homoeostasis and glucose- or KCl-stimulated insulin secretion (Fig. 1), GSIS was only slightly reduced and glucose tolerance remained largely normal in β-barr2-KO mice consuming standard chow (Fig. 4a,b). One possible explanation for the discrepancy between the *in vitro* and *in vivo* findings is that other factors, such as islet innervation, hormonal stimulation of β-cells, or the involvement of non-β-cell pathways allow β-barr2-KO mice to maintain normal glucose homoeostasis when maintained on RC. The identity of these pathways remains to be elucidated.

In contrast, when mice were fed a calorie-rich HFD, *in vivo* studies showed that GSIS was virtually abolished in β-barr2-KO mice (Fig. 4d). Consistent with this finding, HFD β-barr2-KO mice showed a striking exacerbation of glucose intolerance (Fig. 4e) and greatly increased fed and fasting blood glucose levels. One possible explanation for the observation that β-cell barr2 deficiency causes pronounced metabolic deficits only in HFD mice is that barr2-independent signalling pathways are able to maintain normal β-cell function in healthy islets *in vivo* (RC mice). When β-cell function is compromised by the glucolipotoxic effects of a long-term HFD, these barr2-independent signalling pathways are probably no longer able to compensate for the deficits in β-cell function caused by the lack of barr2. Interestingly, mice lacking the exchange protein activated by cAMP islet/brain isoform 2A (EPAC2A KO mice) showed diet-dependent *in vivo* metabolic changes similar to those displayed by the β-barr2-KO mice^45^.

Two recent reports described the function of pancreatic islets prepared from whole-body barr2-KO mice that lacked barr2 throughout development^16^,^17^. In contrast to our findings, Ravier *et al*.^16^ reported that glucose-stimulated increases in insulin release and [Ca^2+^]_i_ were unchanged in islets prepared from whole-body barr2-KO mice. Zhang *et al*.^17^ demonstrated that GSIS was reduced in islets from whole-body barr2-KO mice in a static islet incubation assay, similar to our findings with perifused β-barr2-KO islets. This deficit in GSIS was associated with a reduction in pre-docked insulin vesicles. However, we found that the total number and the density of pre-docked insulin granules were similar in β-barr2-KO and control islets (Supplementary Figs 5 and 6). The most likely explanation for these discrepant observations is that the inactivation of *barr2* in essentially all body cells affects β-cell function in an indirect fashion and that the absence of barr2 during development is likely to trigger unpredictable changes in β-cell function and glucose homoeostasis.

The two β-arrestins, barr1 and barr2, share a high degree of sequence identity (78% at the amino acid level) and act in a similar fashion in many but not all experimental systems^41^. Sonoda *et al*.^46^ demonstrated that barr1 knockdown in cultured β-cells (INS-1 cells) led to a marked decrease in GLP1 receptor-mediated insulin secretion, implicating barr1 in the regulation of insulin secretion. Clearly, studies with conditional, β-cell-specific β-barr1-KO mice are needed to explore the potential role of barr1 in regulating β-cell function and whole-body glucose homoeostasis under *in vivo* conditions.

In patients with T2D and obese individuals, plasma-free fatty acid levels are usually elevated, contributing to impaired β-cell function including reduced GSIS^47^,^48^. Interestingly, we showed that exposure of isolated human pancreatic islets to glucolipotoxic conditions that impair β-cell function led to significant reductions in *BARR2* and *BARR1* expression levels (Fig. 9h,i). This finding, together with our observation that *barr2* knockdown impairs GSIS in both mouse and human β-cells (Fig. 1), suggests that strategies that promote barr2 expression and/or function in β-cells may prove beneficial for restoring impaired β-cell function. Consistent with this notion, pancreatic islets prepared from HFD mice showed reduced *barr2* levels (Fig. 9g) and barr2 overexpression in β-cells greatly ameliorated obesity-associated metabolic deficits (Fig. 9d,e).

In conclusion, we report the novel finding that barr2 is required for the proper function of pancreatic β-cells *in vitro* and *in vivo*. Several lines of evidence indicate that barr2-dependent activation of CAMKII represents the key mechanism through which barr2 exerts its beneficial effects on insulin release and Ca^2+^ entry into β-cells. These findings suggest the intriguing possibility that modulating barr2 function in β-cells may represent a potential new therapeutic approach for the treatment of T2D.

## Methods

### Generation of mice lacking barr2 in pancreatic β-cells

The generation of homozygous floxed *barr2* mice (*fl/fl barr2* mice) in which exon 2 is flanked by loxP sites is described in a separate publication^21^. These floxed mice were obtained on a pure C57BL/6J background. *Pdx1-Cre-ER*^*TM*^ mice with a mixed genetic background were kindly provided by Dr. Doug Melton (Harvard University). We backcrossed the *Pdx1-Cre-ER*^*TM*^ mice for 10 generations onto a C57BL/6 background. Subsequently, we intermated the backcrossed *Pdx1-Cre-ER*^*TM*^ mice with the *fl/fl barr2* mice to generate *fl/fl barr2*-*Pdx1-Cre-ER*^*TM*^ mice and *fl/fl barr2* control littermates. When these mice were 8 weeks old, we injected them for 6 consecutive days with TMX (Sigma) suspended in corn oil (Sigma) (1 mg i.p. per mouse per day). All animals used were maintained on a C57BL/6 background.

All experiments were conducted according to the US National Institutes of Health Guidelines for Animal Research and were approved by the NIDDK Institutional Animal Care and Use Committee.

### Generation of mice overexpressing barr2 in β-cells

An HA-tagged version of rat *barr2* (Addgene) was used to generate a transgene construct, in which the expression of *barr2* was under the transcriptional control of the *RIPII* promoter^28^,^49^. The resulting 4.1 kb transgene was isolated, purified, and microinjected into the pronuclei of ova prepared from C57BL/6 mice (Taconic)^28^. *RIPII-barr2* transgenic mice were identified via PCR analysis of tail DNA (see the next paragraph). All mice were maintained on a pure C57BL/6NTac background.

### PCR genotyping of mutant mice

Floxed *barr2* mice: The following PCR primer pair flanking exon 2 was used to distinguish between the wt *barr2* allele and the floxed *barr2* allele: forward primer, 5′-GAGTCACTGTATGGGTCCCTG-3′; reverse primer, 5′-TTGCTGTTCGATGCTACATAACTC-3′. The use of these primers results in PCR products of the following sizes: wt allele, 349 bp; floxed allele, 507 bp.

To detect the *Pdx1-Cre-ER*^*TM*^ transgene, we used the following PCR primers (size of PCR product, 400 bp): forward primer, 5′-CCTGGAAAATGCTTCTGTCCG-3′; reverse primer, 5′-CAGGGTGTTATAAGCAATCCC-3′.

*RIPII-barr2* transgenic mice: To detect the *RIPII-barr2* transgene, we used the following PCR primers (size of PCR product, 618 bp): forward primer, 5′-AGTTCTGCTACCCATACGAC-3′; reverse primer, 5′-CTCCTTAGTCTCCTCCTCTTAT-3′. The following PCR cycling conditions were used: 95 °C for 5 min followed by 35 cycles at 95 °C for 30 s, 55 °C for 30 s, and 72 °C for 1 min.

### Mouse maintenance and diet

Mice were fed *ad libitum* and kept on a 12-hr light, 12-hr dark cycle. Unless stated otherwise, all experiments were carried out with male littermates that were 10–20 weeks old and maintained on a standard mouse chow (4% (w/w) fat content; Zeigler). In a subset of experiments, 8–10-week-old male mice were switched to a HFD (35.5 % (w/w) fat content; # F3282, Bioserv) for at least 8 weeks.

### Morphometric analysis of pancreatic islets

Pancreata were fixed overnight in 4% paraformaldehyde/phosphate-buffered saline, and embedded in paraffin. For each pancreas, three consecutive 5 μm-thick sections from six distinct levels, 150 μm apart, were mounted on slides and subjected to standard hematoxylin/eosin staining. To determine β-cell mass, we used three mice per genotype (5-month-old males). Six whole pancreatic sections per animal (150 μm apart) were blocked with normal goat serum for 1 h, and incubated overnight at 4 °C with a guinea pig anti-insulin antibody (Thermo Scientific, PA1-26938, dilution 1:100) and a rabbit anti-glucagon antibody (Thermo Scientific, RB-1422-A1, dilution 1:100). The two primary antibodies were detected with Alexa Fluor 555 goat anti-guinea pig (red colour, Invitrogen, A21435, dilution 1:500) or Alexa Fluor 488 goat anti-rabbit (green colour, Invitrogen, A11034, dilution 1:500), respectively. All sections were counterstained with DAPI (Vectashield mounting medium with DAPI, Vector Laboratories) to visualize nuclei (blue colour). For β-cell mass analysis, slides were imaged on a Keyence digital microscope (BZ-9000) with a lens CFI Plan Apo λ 4 × attached. Image acquisition and measurement of total pancreatic and β-cell-specific area for each section were performed using BZ-II Viewer and BZ-II Analyzer software (version 2.1; Keyence). To determine β-cell mass, we calculated the ratio of islet cross-sectional area to total pancreatic area multiplied by pancreatic weight.

### Drugs used for insulin release studies

Drugs employed for insulin release studies were purchased from Tocris Bioscience (nifedipine, FPL64176, oxotremorine-M, exendin-4, veratridine), Sigma (GLP-1) or Millipore (AIP2). The Drosophila homeoprotein Antennapaedia leader peptide (RQIKIWFQNRRMKWKK) was obtained from GenScript.

### Mouse Islet perifusion studies

Islets were isolated from pancreata of control and mutant mice after collagen digestion^50^. Briefly, the pancreatic duct was cannulated with a 27-G needle connected to a 5 cc syringe, and the pancreas was injected with 3–5 ml of cold (4 °C) enzyme solution (HBSS with 25 mM Hepes buffer and collagenase Type V; Sigma). The pancreas was removed and digested at 37 °C for 18 min. Enzymatic activity was terminated by the addition of cold (4 °C) RPMI (10%). Islets were then isolated by spinning, decanting the supernatant, re-suspending and filtering through a 450-μm screen. Finally, islets were purified by Euro-Ficoll density gradient centrifugation. Dynamic measurements of insulin secretion were performed by using a high-capacity, automated islet perifusion system (Biorep Perifusion V2.0.0) (refs 51, 52). A low pulsatility peristaltic pump pushed HEPES-buffered solution (composition in mM: 125 NaCl, 5.9 KCl, 2.56 CaCl_2_, 1 MgCl_2_, 25 HEPES and 0.1% BSA; pH 7.4) at a perifusion rate of 100 μl min^−1^ through a column containing 100 pancreatic islets immobilized in Bio-Gel P-4 Gel (BioRad). In all perifusion experiments, basal glucose concentrations were adjusted to 3 mM. Stimuli (16 mM glucose for 15 min or 25 mM KCl for 5 min, respectively) were applied with the perifusion buffer. The perifusate was collected in an automatic fraction collector designed for a 96-well plate format. The columns containing the islets and the perifusion solutions were kept at 37 °C, and the perifusate in the collecting plate was kept at <4 °C. Perifusates were collected every minute. Insulin release in the perifusate was determined with an ultra-sensitive mouse insulin Elisa kit (Mercodia). DNA extracted from islets was quantified using the PicoGreen kit (Invitrogen) and used for normalization of islet size and number. We calculated the area under the curve (AUC) over the 15-min stimulation period with high glucose or the 5-min KCl stimulation period to determine the total amount of insulin secreted during these stimulatory conditions.

### Cytoplasmic Ca^2+^ measurements

To determine cytoplasmic Ca^2+^ concentrations ([Ca^2+^]_i_), we used a Fura-2-based imaging procedure^51^,^52^. Mouse pancreatic islets were immersed in HEPES-buffered solution. Glucose was added to a final concentration of 3 mM. Islets were incubated in Fura-2 AM (2 μM) for 1 h and placed in a small-volume imaging chamber (Warner Instruments). Stimuli were applied with the bathing solution. Islets loaded with Fura-2 were alternatively excited at 340 and 380 nm with a monochromator light source (Cairn Research Optoscan Monochromator, Cairn Research Ltd, Faversham, UK). Images were acquired with a Hamamatsu camera (Hamamatsu Corp., Japan) attached to a Zeiss Axiovert 200 microscope (Carl Zeiss, Jena, Germany). Changes in the 340/380 nm fluorescence emission ratio after addition of high glucose (16 mM) or KCl (25 mM) were analysed over time in individual islets using MetaFluor imaging software. Peak changes in the fluorescence ratio were measured to compare response profiles between islets (delta [Ca^2+^]_i_=(maximum 340/380 value)−(baseline 340/380 value)). In all experiments, the basal concentration of glucose was 3 mM.

### Determination of pancreatic insulin content

Total pancreatic insulin content was measured by using an acid-ethanol method^53^.

### Immunohistochemistry and imaging studies (mouse islets)

Immunohistochemical stains were performed using standard procedures. Fluorescent images were captured and processed under identical parameters with a Zeiss Imager D1 fluorescent microscope. Confocal images were obtained and processed using an LSM 700 confocal microscope with a 20 × /0.45 N-Achroplan objective (Zeiss, Germany). The antibodies used are included in Supplementary Table 2.

### Western blotting studies

Western blotting studies were carried out with lysates prepared from MIN6 cells or isolated pancreatic islets by using standard techniques^54^. Protein bands were quantitated by using ImageJ software (NIH). The antibodies used are listed in Supplementary Table 2. Original uncropped blots are presented in Supplementary Figs 16 and 17.

### Determination of gene expression levels via qRT-PCR

Gene expression levels were measured via qRT-PCR using total RNA prepared from different mouse tissues, human pancreatic islets or cultured MIN6 cells. The PCR primers used for qRT-PCR experiments are listed in Supplementary Table 3.

### Perforated patch electrophysiology

β-Cells on the periphery of islets were sealed in voltage clamp at −80 mV (>2 gΩ seal). Patch electrodes were pulled with tip resistance between 3–4 μΩ and loaded with intracellular solution containing (in mM) 140 KCl, 1 MgCl_2_[H_2_O]_6_, 10 EGTA, 10 HEPES (pH 7.25 adjusted with KOH) and the pore-forming antibiotic amphotericin B (Sigma)^55^. After perforation of the plasma membrane by amphotericin B, the cell was transitioned to current clamp mode^55^. Islets were treated with 3 or 16 mM glucose dissolved in Krebs-Ringer-buffer (KRB) of the following composition (in mM): 119 NaCl, 2 CaCl_2_, 4.7 KCl, 10 HEPES, 1.2 MgSO_4_ and 1.2 KH_2_PO_4_ (pH 7.35 adjusted with NaOH). The starting glucose concentration was 3 mM and was followed by 16 mM glucose for 10–20 min. The frequency of AP firing in response to 16 mM glucose was determined^56^.

### Whole-cell voltage clamp electrophysiological recordings

VDCCs, K_ATP_ and K_V_ currents were recorded from dispersed, primary mouse β-cells with the whole-cell voltage clamp technique^31^. Patch electrodes were pulled with tip resistance between 3–4 μΩ. For VDCC recordings, patch electrodes were loaded with intracellular solution containing (in mM): 102 CsCl, 10 TEA chloride, 0.1 tolbutamide, 10 EGTA, 1 MgCl_2_, 3 Na_2_ATP, 5 HEPES, pH 7.4, adjusted with CsOH. Whole β-cell seals (>1 giga-ohm) were made in KRB buffer in the presence of 3 mM glucose followed by perifusion with a modified, high Ca^2+^-containing KRB buffer of the following composition (in mM): 82 NaCl, 20 TEA chloride, 0.1 tolbutamide, 30 CaCl_2_, 5 CsCl, 1 MgCl_2_, 0.1 EGTA, 10 glucose, 5 HEPES, pH 7.4, adjusted with NaOH. After perfusion of β-cells for 3 min with this buffer, the first voltage step recording protocol was initiated. Subsequently, K_V_ and K_ATP_ voltage clamp recordings were performed and analysed using Clampfit software (Molecular Devices)^31^.

### Preparation of islets for electron microscopic studies

Male mice (age: 2–3 months) were anaesthetized with ketamine (50 mg kg^−1^ i.p.). Collagenase solution (Sigma) was injected into the bile duct to inflate the pancreas. After digestion, islets were manually selected and washed in Krebs-Ringer HEPES buffer, and cultured overnight in RPMI-1640 medium (Invitrogen) before further experiments^57^,^58^.

To prepare specimens for SBF-SEM, a heavy metal staining procedure was used to fix and stain islets^59^. After staining, the samples were dehydrated and embedded in Epon-Araldite according to standard protocols.

To minimize specimen charging by the electron beam of the SEM, each epon-embedded islet was mounted on an empty resin block and trimmed under a microtome until the stained islet became exposed in the block. The block was subsequently remounted by gluing the exposed face downwards on an aluminium specimen pin (Gatan) using CircuitWorks Conductive Epoxy (CW2400) so that the tissue could maintain electrical contact with the pin. Each specimen block was then trimmed again to expose the opposite side of the islet, and coated with 40–100 nm gold using a Quorum Q150RS sputter coater (EM Sciences).

### SBF-SEM

The trimmed, epon-embedded, stained blocks were imaged using a Gatan 3View serial block-face imaging system installed on a Zeiss SIGMA-VP (variable pressure) SEM. The SEM was operated in high-vacuum mode, with an accelerating voltage of 1.5 kV and a standard 30 μm condenser aperture. Block-face images were collected with a pixel size of 9.9 nm in the *x*–*y* plane with removal of 50 nm slices along the *z* axis, and a pixel dwell time of 1.5 μs. The resulting image series were aligned using Digital Micrograph software (Gatan) and uploaded to Amira software (FEI Inc.) for three-dimensional (3D) visualization and quantitative analysis of individual beta cells from each genotype.

### Determination of the total number of dense core vesicles

The total number of DCVs per β-cell was estimated by utilizing a combination of 3D volume measurements and two-dimensional vesicle density measurements^60^. The cellular, nuclear and mitochondrial volumes per β-cell were measured using Amira software, after which the nuclear and mitochondrial volumes were subtracted from the cell volume to determine the volume available to DCVs. To determine the average vesicle density in each β-cell, five vesicle-rich squares of size 2 μm × 2 μm were extracted from SBF-SEM planes at roughly even intervals throughout each cell. In each square, the number of DCVs per unit area *n*_gran_ was determined and the average *n*_*gran*_ was used to calculate the number of DCVs per unit volume *ρ*_gran_ by using equation (1):


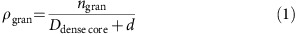


where *D*_dense core_ is the average diameter of the vesicle's dense core, and *d* is the slice thickness^61^. The average DCV dense core diameter has been previously measured to be 240±42 nm (ref. 61). Measurements of ρgran were multiplied with available DCV volumes to yield estimates of the total DCV number in each β-cell.

### Measurement of the density of docked DCVs

The density of DCVs docked at the plasma membrane in each β-cell was determined by first selecting a single region along the cell membrane with a surface area of at least 30 μm^2^. Surface areas of selected regions were computed in Digital Micrograph using the software's measurement tools to determine the width and height of the region. Successive slices throughout the 3D data set were then examined to manually count the number of DCVs in direct contact with the membrane at any instance in the selected region.

### Physiological studies

*In vivo* metabolic tests were performed using standard procedures. In brief, to measure glucose tolerance (IGTT), mice were fasted overnight for 12 h. Blood glucose concentrations were determined using blood collected from the tail vein immediately before and 15, 30, 60, 90 and 120 min after i.p. injection of glucose (RC, 2 g kg^−1^; HFD, 1 g kg^−1^). To obtain a measure of peripheral insulin sensitivity (ITT), mice were fasted for 4 h, and blood glucose concentrations were measured before and at the indicated time points after i.p. injection of human insulin (RC, 0.75 U kg^−1^; HFD, 1 U kg^−1^; Novo Nordisk). Blood glucose levels were determined using an automated blood glucose reader (Glucometer Elite Sensor; Bayer). To study glucose-stimulated insulin secretion (GSIS), mice were fasted overnight for 12 h, and then injected with the same doses of glucose (i.p.) as used for the IGTT. Blood samples were collected from the tail vein at different time points (0, 5, 15 and 30 min) and centrifuged for plasma collection (3,000 g, 5 min, 4 °C). Plasma insulin concentrations were determined by using an ELISA kit (Crystal Chem Inc.).

### Measurement of CAMKII activity in mouse pancreatic islets

CAMKII activity assays were carried out with isolated mouse pancreatic islets^62^. In brief, islets (200–300 per tube) were pre-incubated in KRBH buffer with 2.8 mM glucose for 15 min at 37 °C, and then transferred to either low-glucose (2.8 mM) or high-glucose (28 mM) KRBH buffer. After a 2.5 min incubation period, islets were sonicated on ice with 50 μl of lysis buffer containing 20 mM Tris/HCl, pH 8.0, 2 mM EGTA, 2 mM EDTA, 10 μg ml^−1^ aprotinin, 2 mM DTT, 1 mM PMSF, and proteinase and phosphorylase inhibitors (Roche). CAMKII activity was determined with a widely used assay kit (SignaTECT Calcium/Calmodulin-Dependent Protein Kinase Assay System, V8161; Promega). This kit involves the use of biotinylated CAMKII peptide substrate to determine CAMKII-dependent phosphorylation. Phosphorylation reactions were initiated by mixing 5 μl of islet homogenate with 25 μl of a solution containing (final concentrations) 50 mM Tris-HCl (pH 7.5), 10 mM MgCl_2_, 0.5 mM DTT, 0.1 mM ATP, 0.5 μCi [γ^32^P]-ATP (3000 Ci per mmol, 10 mCi per ml; PerkinElmer), and biotinylated substrate (concentration: 50 μM). Total CAMKII activity was determined in the presence of 1 mM CaCl_2_ and 1 μM calmodulin. Ca^2+^-independent, autonomous CAMKII activity was assessed in the presence of 1 mM EGTA and the absence of CaCl_2_/calmodulin. After a 2 min incubation period at 30 °C, the reaction was terminated by the addition of guanidine hydrochloride (final concentration: 2.5 M). Following a centrifugation step at 12,000*g* for 1 min, 10 μl of supernatant was spotted onto a pre-numbered square of membrane impregnated with streptavidin. Membranes were washed and dried according to the manufacturer's instructions. The amount of ^32^P bound to the membrane squares was measured via a liquid scintillation counting. CAMKII activities were normalized to the amount of protein contained in each sample.

### Insulin release studies with cultured β-cells (MIN6 cells)

MIN6 cells were a kind gift from Dr. Abner Notkins (NIDCR, NIH) (original source: Dr J. Miyazaki, University of Osaka, Japan). For gene silencing studies, ∼1 × 10^6^ MIN6 cells were electroporated with 100 pmoles of mouse *barr2* siRNA or negative control siRNA (SMARTpool siRNA; Dharmacon), according to the manufacturer's instructions (Dharmacon). In a subset of experiments, cells were infected with the KD-CAMKIIδ or CA-CAMKIIδ adenoviruses (100 MOI (multiplicity of infection)) ∼24 h after electroporation^34^. About 48 h after electroporation, insulin secretion studies were carried out with transfected/infected MIN6 cells grown in six-well plates^63^. In brief, cells were pre-incubated with Krebs-Ringer HEPES (KRBH) buffer for 30 min, and then switched to either low-glucose (2.8 mM) or high-glucose (16.7 mM) KRBH buffer. KCl-induced insulin secretion was monitored via incubation of MIN6 cells with KRBH buffer containing 30 mM KCl (1 h at 37 °C; 2.8 mM glucose). The amount of insulin released into the medium was measured by using an ELISA kit (Crystal Chem Inc.).

### Adenoviral transduction of mouse pancreatic islets

Mouse pancreatic islets were isolated and transduced with adenoviruses using a published procedure^64^, with minor modifications. In brief, islets were isolated from 10- to 16-week-old male mice by collagenase digestion and Histopaque gradient purification. Islets were then immediately transduced at an MOI of 100 with either Ad-GFP or Ad-CA-CAMKIIδ for overnight at 37 °C. After washing, islets were incubated for another 24 h in RPMI 1640 medium at 37 °C in the presence of 5% CO_2_. For insulin secretion assays, islets were first pre-incubated with KRBH buffer for 30 min and then switched to either low-glucose (2.8 mM) or high-glucose (28 mM) KRBH buffer, followed by a 1 h incubation at 37 °C. Insulin measurements were carried out as described in the previous paragraph.

### Insulin secretion studies with human EndoC-βH1 cells

EndoC-βH1 cells^25^ were a kind gift by Dr Raphaël Scharfmann (INSERM U1016, Institut Cochin, Université Paris Descartes, France). Briefly, EndoC-βH1 cells were cultured in a humidified incubator at 37 °C, 5% CO_2_, with DMEM (Life Technologies) containing 1 mg ml^−1^ glucose, 2% albumin from bovine serum fraction V (Equitech), 50 μM 2-mercaptoethanol (Sigma), 10 mM nicotinamide (VWR), 5.5 μg ml^−1^ transferrin (Sigma), 6.7 ng ml^−1^ sodium selenite (Sigma) and penicillin (100 units ml^−1^)/streptomycin (100 μg ml^−1^). The culture supports were pre-coated with medium containing ECM (1%; Sigma) and fibronectin (2 μg ml^−1^; Sigma). Cells were passaged every 5–7 days and maintained at a density of ∼6 × 10^6^ cells per 10 cm^2^ dish. For gene silencing studies, ∼1 × 10^6^ cells were electroporated with 100 pmoles of human *barr2* siRNA or scrambled control siRNA (SMARTpool siRNA; Dharmacon), according to the manufacturer's instructions (Dharmacon). Two days after plating of the cells, the medium was replaced with DMEM without glucose, and cells were allowed to continue to grow overnight. Insulin secretion studies were then carried out using the same strategy as described above for murine MIN6 cells. In brief, cells were pre-incubated with Krebs-Ringer HEPES (KRBH) buffer with 0.5 mM glucose for 1 h, and then switched to same KRBH buffer with different concentrations of glucose (0.5 mM or 25 mM, respectively) containing 500 μM IBMX for 1 h at 37 °C. Insulin concentrations were measured by using a human insulin ELISA kit (Alpco Inc.).

### Human pancreatic islets

Human pancreatic islets were received from the accredited Human Islet Resource Center at the University of Pennsylvania. Islets were obtained from six individuals, who were normoglycemic at the time of organ isolation. Human islets were then cultured and transferred to the laboratory^65^,^66^ . The University of Pennsylvania Institutional Review Board has exempted this research from ethical review because the islets were received from deceased, de-identified organ donors. All pancreata acquired by the Human Islet Resource Center at the University of Pennsylvania were from deceased donors after having obtained consent from their families through UNOS (United Network for Organ Sharing).

### Preparation of fatty acid solutions

A 5 mM stock solution of sodium palmitate (Sigma-Aldrich) was prepared by dissolving the fatty acid salt in 10% BSA (Sigma-Aldrich, fraction V, fatty acid-free) in Krebs buffer by continuous stirring for ∼4 h in a 37 °C water bath. The stock solution was then diluted by Krebs buffer to obtain the final concentration of 0.5 mM sodium palmitate. To prepare a 2:1 mixture of palmitate and oleate (final fatty acid concentration: 0.5 mM), sodium palmitate was dissolved first, followed by the addition of the proper amount of sodium oleate (Sigma-Aldrich).

### Perifusion of human islets for insulin release measurements

Handpicked human islets (180 islets per group) were placed on a nylon filter and perfused at a flow rate of 1.2 ml min^−1^ in a plastic perifusion chamber (Millipore)^67^. The perifusion apparatus consisted of a computer-controlled low pressure chromatography system (BIO-RAD Econo system) with programmable rates of flow and glucose concentrations in the perfusate, a 37 °C water bath, and a fraction collector (BIO-RAD; model 2128). The perifusion solution was a Krebs buffer (pH 7.4) containing (in mM): 114 NaCl, 5 KCl, 24 NaHCO_3_, 1 MgCl_2_ 6H_2_O, 2.2 Ca^2+^, 1 Pi, 10 HEPES (pH 7.4) and 1% of BSA (Sigma-Aldrich: fraction V, fatty acid-free) equilibrated with 20% O_2_ and 5% CO_2_ balanced with N_2_. Insulin secretion was initiated by incubating islets consecutively with 4 and 8 mM glucose. We applied these rather low-glucose concentrations since human islets are far more sensitive to glucose than mouse or rat islets (10 mM glucose causes a maximum insulin response in human islets^67^). The AUC for glucose (4 or 8 mM)-stimulated insulin release was calculated using GraphPad software (Prism 6.0.)

qRT-PCR studies with total human islet RNA were carried out in the same fashion as described above for mouse islet RNA.

### Co-immunoprecipitation of endogenous barr2/CAMKII complexes

Wt islets (∼1,000) were lysed in co-IP lysis buffer containing 20 mM Tris ·HCl (pH 8.0), 137 mM NaCl, 2 mM EDTA, 1% Nonidet P-40, and a mixture of protease and phosphatase inhibitors (Roche). After centrifugation (16,000*g* at 4 °C for 10 min), the supernatant was divided into two aliquots. Immunoprecipitation studies were performed as described^68^. In brief, samples were incubated overnight at 4 °C with either an anti-CaMKIIδ antibody (goat IgG, Santa Cruz) or the same amount of goat IgG (Santa Cruz). After this step, reaction mixtures were incubated with protein A/G PLUS agarose beads (Santa Cruz) for 4 h at 4 °C. The immunoprecipitated proteins were washed three times with a buffer containing 10 mM Tris ·HCl (pH 7.4), 150 mM NaCl, 1 mM EDTA, 1 mM EGTA, and 1% Triton X-100, followed by SDS-PAGE analysis.

Immunoblots were probed with anti-barr2, anti-CaMKII _(pan)_, and anti-Ca_V_1.2 (α_1_ (α1 subunit^69^) antibodies, using the Quick Western Kit (IRDye 680RD, LI-COR Biosciences). Western blots were scanned and validated with an infrared imaging system (Odyssey CLx; LI-COR Biosciences). The antibodies used are listed in the Supplementary Table 2 above.

### Co-immunoprecipitation studies using barr2 fragments

HEK293T cells (ATCC, American Type Culture Collection, CRL-3216) grown in 60-mm plates were transfected with expression plasmids (3 μg each) coding for HA-tagged full-length barr2, the N-domain of barr2 (residues 1–181), or the C-domain of barr2 (residues 180–408). All constructs carried an N-terminal HA epitope tag^70^. Subsequently, transfected cells were infected with an adenovirus (100 MOI) coding for flag-CAMKIIδ or an empty control adenovirus (viruses were a kind gift by Dr Harold Singer, Albany Medical College, NY, USA). About 24 h after the addition of virus, cells were lysed and co-immunoprecipitation studies were carried out as described in the previous paragraph. Flag-CAMKIIδ was immunoprecipitated with an anti-flag antibody (anti-flag M2 antibody, Sigma). The HA-tagged barr2 fragments and full-length barr2 were detected via Western blotting using a rabbit anti-HA monoclonal antibody (Cell Signaling).

### MBP pull-down assay

Purified CAMKIIδ (CAMKII) was a kind gift by Dr Andy Hudmon (Indiana University, Indianapolis, Indiana, USA). We followed a pull-down protocol very similar to that described by Zhan *et al*.^39^. In brief, purified MBP-barr2 (ref. 39) or MBP alone (5 μg each) were incubated with purified JNK3 (JNK3α2; positive controlref. 39) or CAMKII (5 μg each) and amylose resin (25 μl; NEB) in 500 μl binding buffer (20 mM Tris-HCl pH 8, 137 mM NaCl, 1% Nonidet P-40, and 2 mM EDTA) supplemented with EDTA-free protease inhibitor (Roche) for 3 h at 4 °C under gentle rotation. Final protein concentrations were: MBP-barr2 and MBP, ∼0.11 μM; JNK3 and CAMKII, ∼0.18 μM. The resin was then washed three times with wash buffer containing 10 mM Tris-HCl pH 7.4, 150 mM NaCl, 1 mM EDTA, 1 mM EGTA, and 1% Triton X-100. Bound proteins were eluted with 100 μl buffer containing 50 mM HEPES pH 7.3, 150 mM NaCl, and 50 mM maltose. Eluates were analysed by SDS-PAGE and Western blotting.

### Statistical analysis

Data are expressed as means±s.e.m. for the indicated number of observations. The statistical tests used are indicated in the figure legends.

### Data availability

The authors declare that all data supporting the findings of this study are available within the paper and its supplementary information files or from the authors upon reasonable requests.

## Additional information

**How to cite this article:** Zhu, L. *et al*. β-arrestin-2 is an essential regulator of pancreatic β-cell function under physiological and pathophysiological conditions. *Nat. Commun.* **8,** 14295 doi: 10.1038/ncomms14295 (2017).

**Publisher's note:** Springer Nature remains neutral with regard to jurisdictional claims in published maps and institutional affiliations.

## Supplementary Material

Supplementary Information Supplementary figures, supplementary tables and supplementary references.
