# Supplementary material for: β-arrestin-2 is an essential regulator of pancreatic β-cell function under physiological and pathophysiological conditions
Source: Nat Commun. 2017 Feb 1;8:14295. doi: 10.1038/ncomms14295 (PMC5296650; doi:10.1038/ncomms14295)
Supplement: Supplementary Information — Supplementary figures, supplementary tables and supplementary references. [file ncomms14295-s1.pdf]

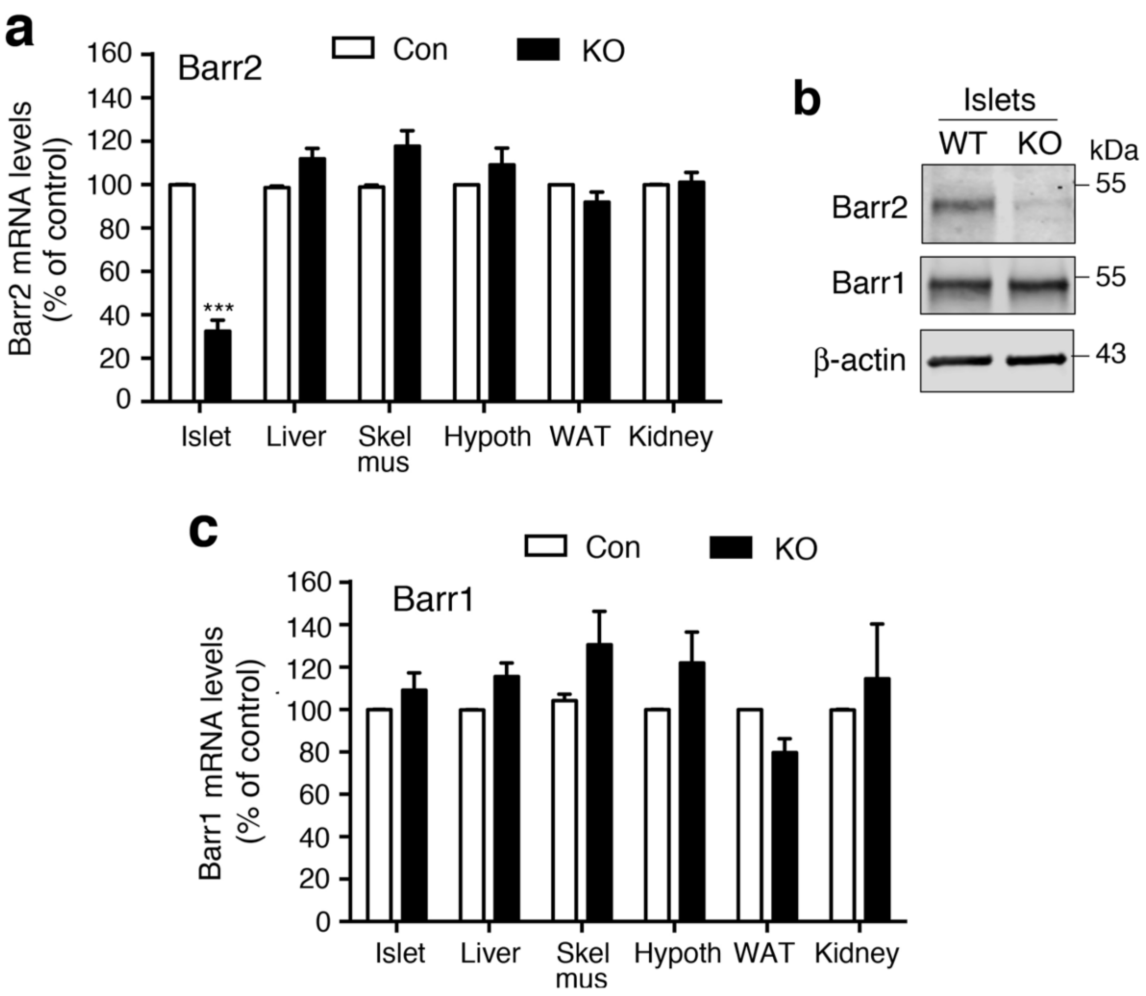

Supplementary Figure 1. Generation of β-cell-specific barr2 knockout mice (β-barr2-KO mice). Floxed *barr2* mice carrying the *Pdx1-Cre-ER*^{TM} transgene (*fl/fl barr2-Pdx1-Cre-ER*^{TM} mice) and their *fl/fl barr2* control littermates (8-week-old males) were injected with TMX for 6 consecutive days, as described under Methods. *Barr2*- and *barr1* expression levels were then examined by qRT-PCR using total RNA prepared from the indicated tissues (for primer sequences, see Methods). Skel mus, skeletal muscle; Hypoth, hypothalamus; WAT, white adipose tissue. (**a**) *Barr2* mRNA expression is selectively reduced in islets from TMX-injected *fl/fl barr2-Pdx1-Cre-ER*^{TM} mice (β-barr2-KO mice). (**b**) Representative Western blots showing greatly reduced barr2 protein expression in islets from β-barr2-KO mice (note that barr1 protein expression is similar in control and KO islets). Equal amounts of islet lysates (150 islets per genotype) were loaded. (**c**) Deletion of *barr2* in β-cells/islets of adult mice has little or no effect on *barr1* transcript levels. RNA expression data are given as means ± s.e.m. (3 mice per genotype). ***$p<0.001$, as compared to the corresponding control group (Student's t-test).

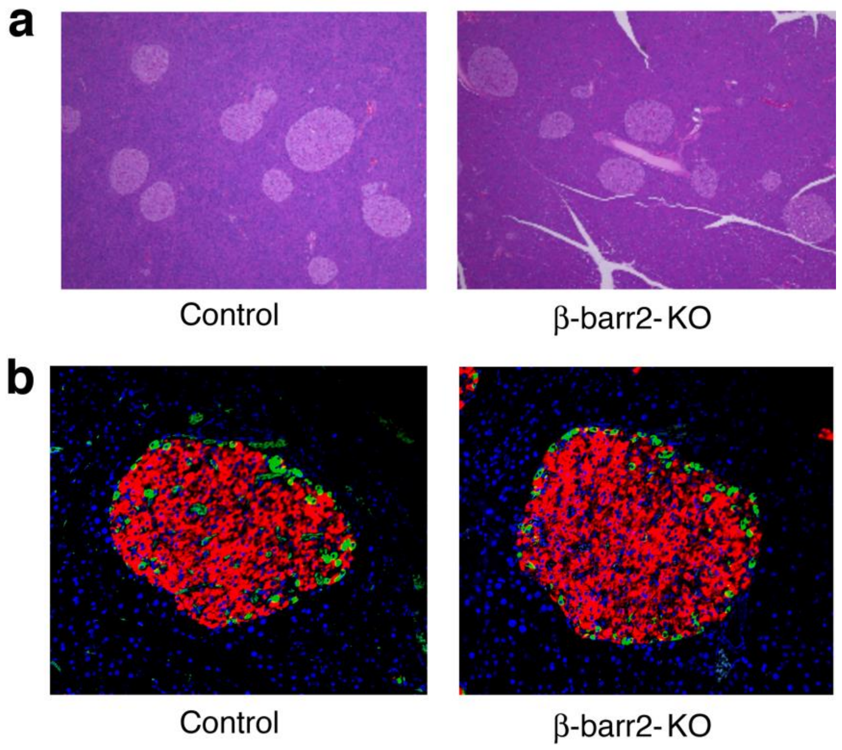

Supplementary Figure 2. Islet morphology remains unaffected by β-cell barr2 deficiency. (**a**) Representative images of pancreatic sections from β-barr2-KO mice and control littermates (H&E staining). (**b**) Representative confocal images of islets from β-barr2-KO mice and control littermates stained with anti-insulin (red) and anti-glucagon (green) antibodies. Samples were prepared from 16-week-old male mice.

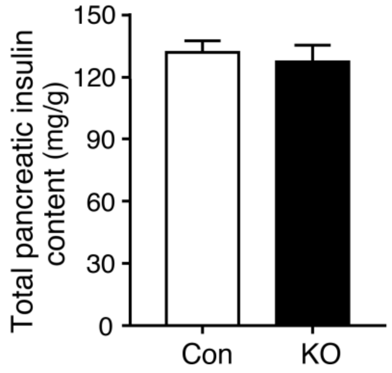

Supplementary Figure 3. β-Cell barr2 deficiency has no effect on pancreatic insulin content. Pancreatic insulin content was similar in β-barr2-KO mice and control littermates (16-week-old males) maintained on regular mouse chow. The two groups did not differ in pancreatic weight: control, 307±8 mg; KO, 319±6 mg. Data are expressed as means ± s.e.m. (n=7-9 per group).

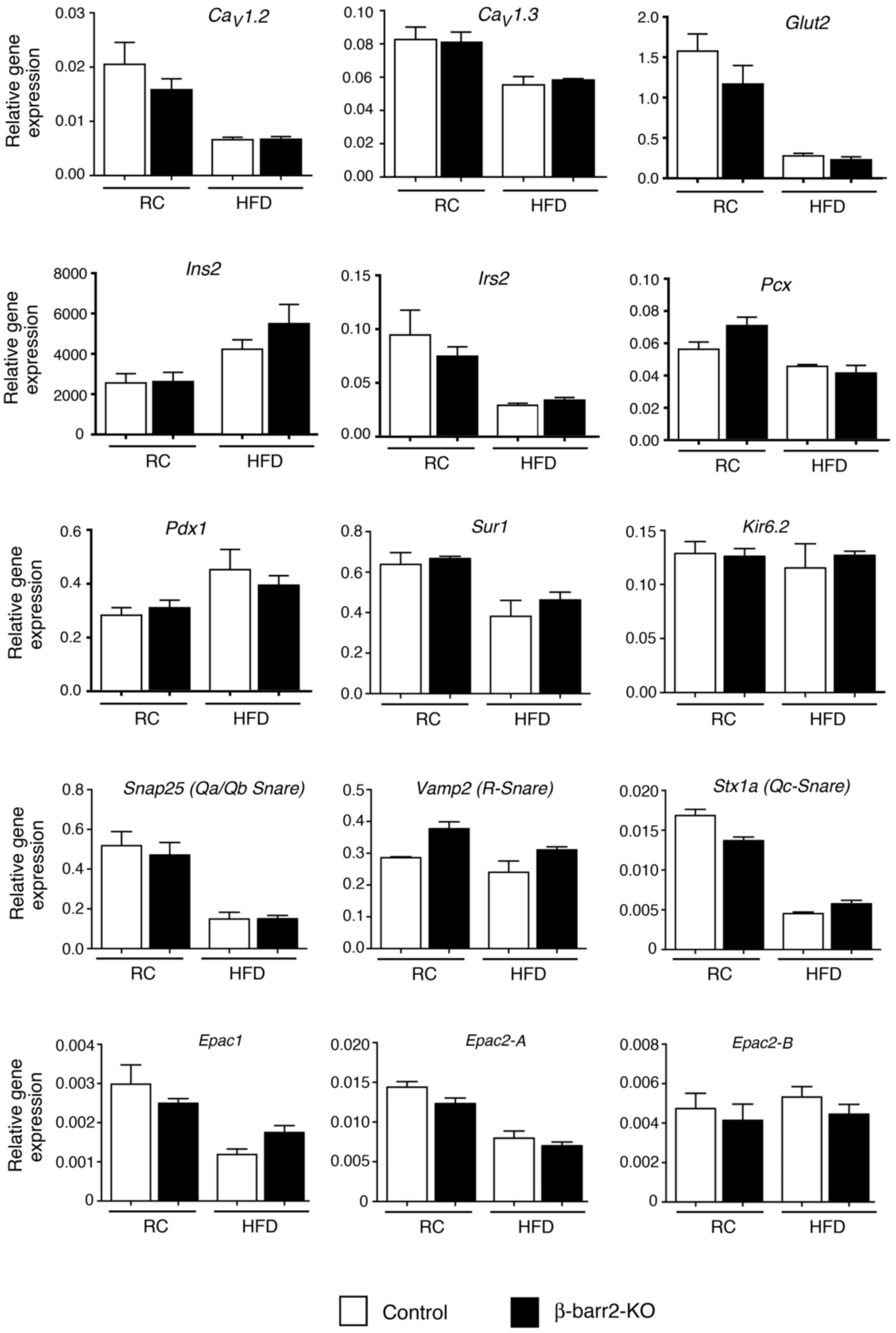

Supplementary Figure 4. β-Cell barr2 deficiency has no effect on the expression levels of key β-cell genes. Total RNA was prepared from pancreatic islets of β-barr2-KO mice and control littermates (16-20-week-old males; 3-5 mice per genotype). Subsequently, gene expression levels were determined via real-time qRT-PCR. Transcript levels were normalized relative to the expression of *β-actin* (for primer sequences, see Methods).

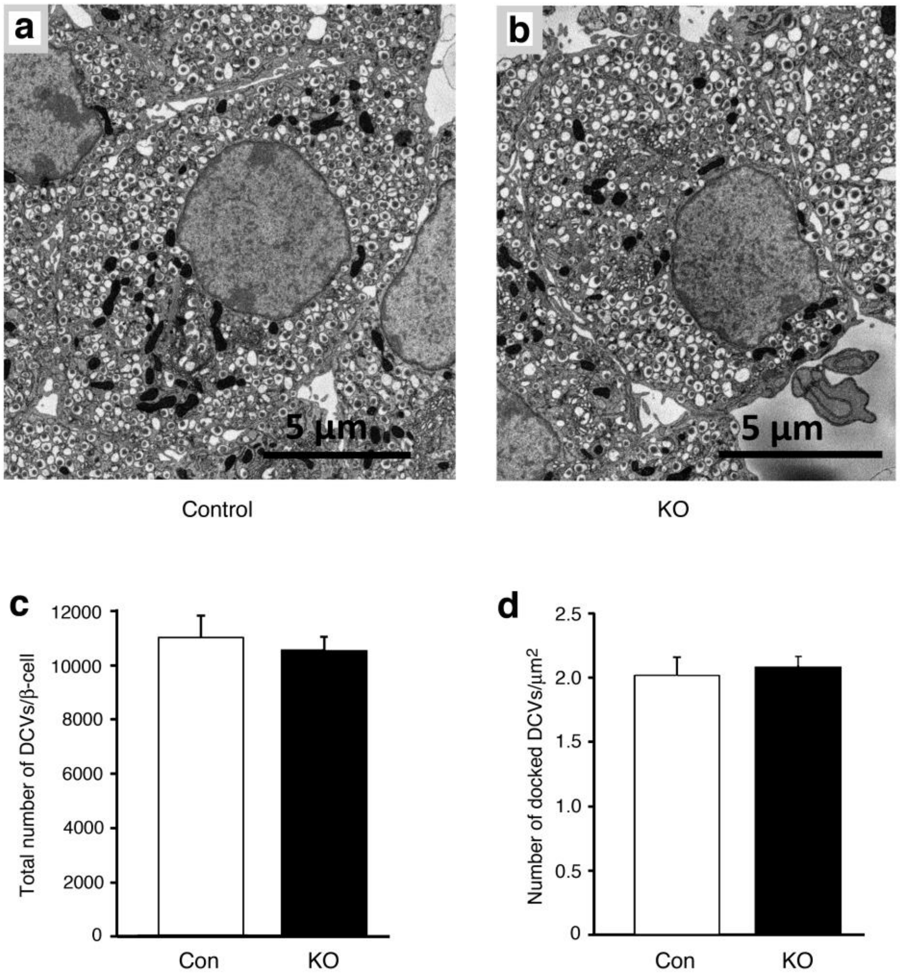

Supplementary Figure 5. Lack of barr2 has no effect on the total number and density of membrane-docked DCVs. (**a, b**) Representative SBF-SEM slices of β-cells from (A) control and (B) β-barr2 KO mice. (**c**) Total number of DCVs in β-cells from control and β-barr2 KO mice. (**d**) Number of DCVs docked to the β-cell plasma membrane in control and β-barr2 KO mice. Data are given as means ± s.e.m. (10-12 β-cells per genotype derived from 2 mice per genotype).

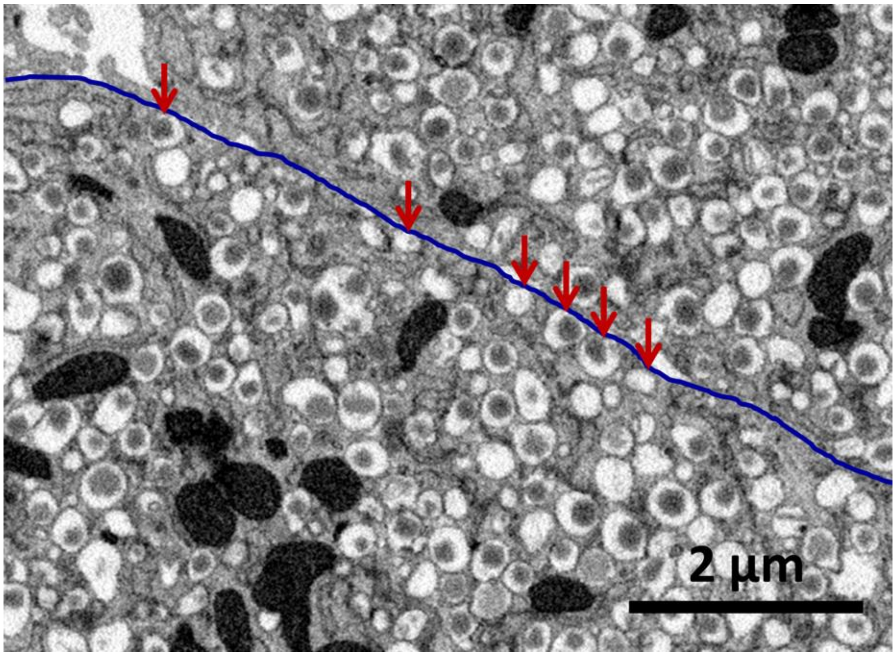

Supplementary Figure 6. Visualization of DCVs docked to the β-cell plasma membrane. A single SBF-SEM 50 nm slice showing the boundary between the membranes of two β-cells (blue). The DCVs that are docked to, or touching the cell membrane are marked with red arrows.

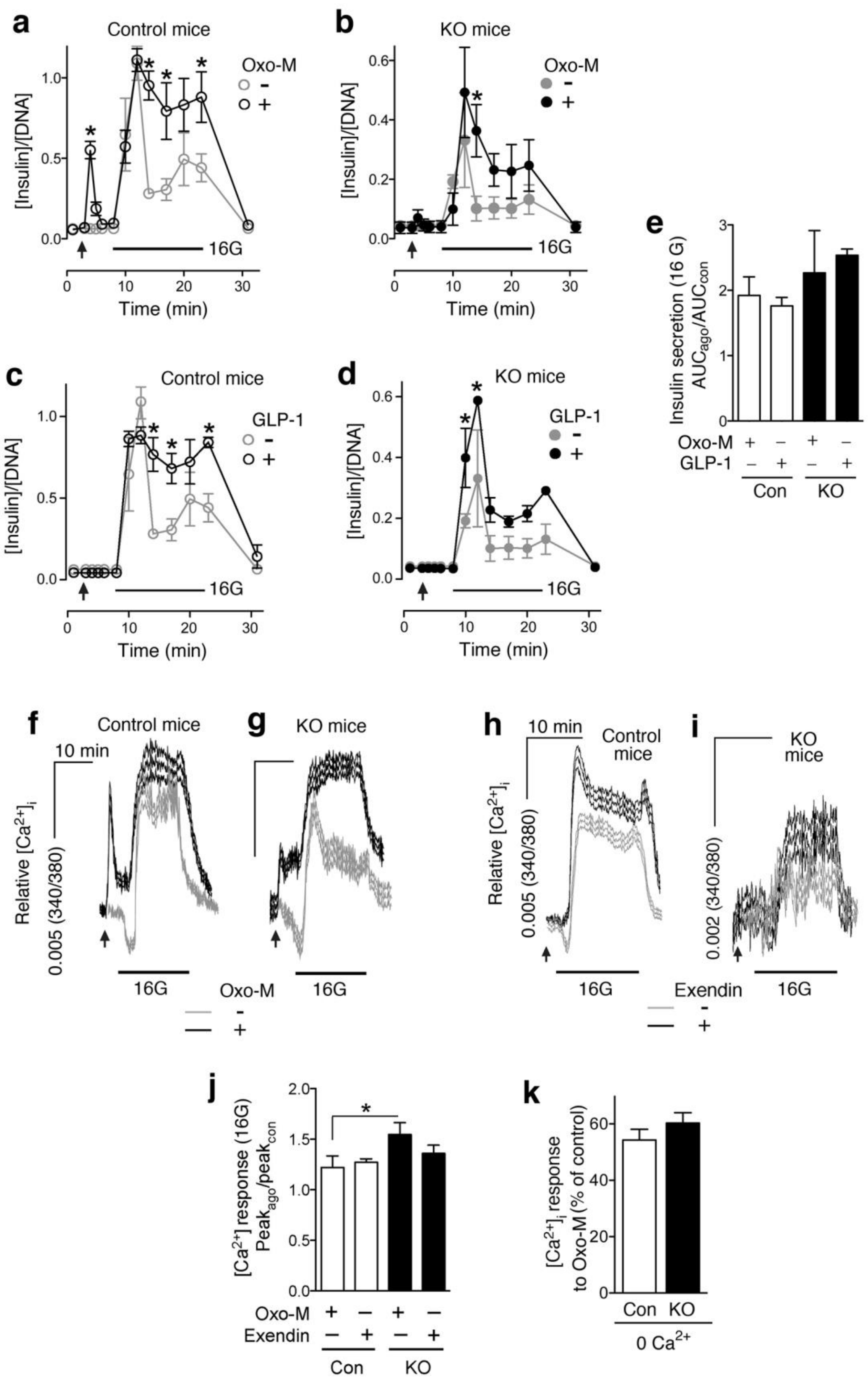

Supplementary Figure 7. GPCR-mediated augmentation of insulin and $[Ca^{2+}]_i$ responses in β-barr2-KO and control islets. (**a-d**) Islet perifusion studies. Oxo-M- (a, b)

and GLP-1 (c, d)-mediated augmentation of glucose (16G)-stimulated insulin release from control (a, c) and β-barr2-KO (b, d) islets. Oxo-M is a muscarinic agonist that facilitates insulin secretion by activating β-cell M_3 muscarinic receptors. Oxo-M (10 μM) or GLP-1 (100 nM) were added 5 min prior to glucose stimulation (indicated by arrows). Please note that the curves generated in the absence of drugs are identical in (a, c) and (b, d), respectively, since all experiments were carried out simultaneously with three types of islets (no drug or Oxo-M or GLP-1 treatment). All data shown represent means ± s.e.m. (n=3 perifusions per condition; islets were isolated from 6 male mice per genotype; *$p<0.05$, two-way repeated measures ANOVA followed by Bonferroni post-tests). (**e**) Quantification of the data presented in (a-d). This plot summarizes 16G-induced insulin secretion in the absence or presence of two GPCR agonists, Oxo-M (10 μM) and GLP-1 (100 nM). The stimulatory effects of the two GPCR agonists on insulin secretion were not statistically different in control and β-barr2-KO islets (one-way ANOVA followed by Tukey's post-test). AUC_{ago} and AUC_{con}, area under the curve in the presence or absence of agonist, respectively. (**f-i**) $[Ca^{2+}]_i$ responses after stimulation of islets with 16G under different experimental conditions. Control and β-barr2-KO islets were stimulated with 16G in the absence or presence of 10 μM Oxo-M (f, g) or in the absence and presence of 50 nM exendin-4 (Exendin) (h, i). The addition of drugs is indicated by arrows. Traces represent average responses with associated s.e.m. from 10 cells, representative of 8 islets per genotype (middle traces represent average responses and upper and lower traces denote s.e.m., respectively). (**j**) Quantification of the data shown in (f-i). Increases in $[Ca^{2+}]_i$ are expressed as ratios of peak $[Ca^{2+}]_i$ responses obtained in the presence or absence of agonists (glucose concentration: 16 mM). Note that β-cell barr2 deficiency did not diminish the ability of Oxo-M and exendin-4 to promote increases in $[Ca^{2+}]_{i.}$ In fact, Oxo-M treatment of β-barr2-KO islets led to a significant elevation of $[Ca^{2+}]_{i,}$ as compared to Oxo-M-treated control islets (one-way ANOVA followed by Tukey's post-test). (**k**) $[Ca^{2+}]_i$ responses to Oxo-M (10 μM) are similar in control and β-barr2-KO islets in the absence of extracellular Ca^{2+} (means ± s.e.m.; n=4 islets per genotype; 100% = control $[Ca^{2+}]_i$ responses in regular 3 mM glucose medium). This observation suggests that receptor-mediated release of Ca^{2+} from intracellular stores remains unaffected by β-cell barr2 deficiency.

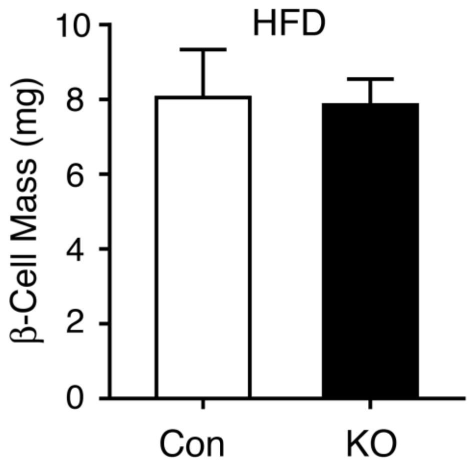

Supplementary Figure 8. β-Cell barr2 deficiency has no effect on β-cell mass in high-fat diet mice. β-Cell mass was similar in β-barr2-KO mice and control littermates (males) maintained on a high-fat diet (HFD) for 16 weeks. The two groups did not differ in pancreatic weight: control, 377±13 mg; KO, 335±17 mg. Data are expressed as means ± s.e.m. (n=3 or 4 per group).

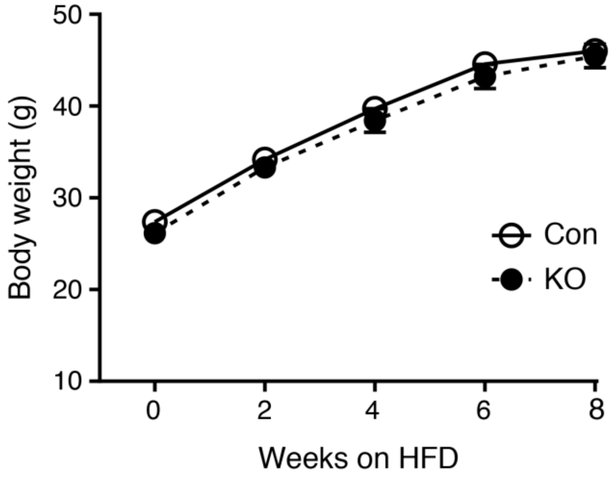

Supplementary Figure 9. β-Cell barr2 deficiency has no effect on body weight gain when mice are maintained on a high-fat diet. β-barr2-KO mice and control littermates (males) were maintained on a high-fat diet (HFD) for 8 weeks. Data are given as means ± s.e.m. (8-12 mice per group).

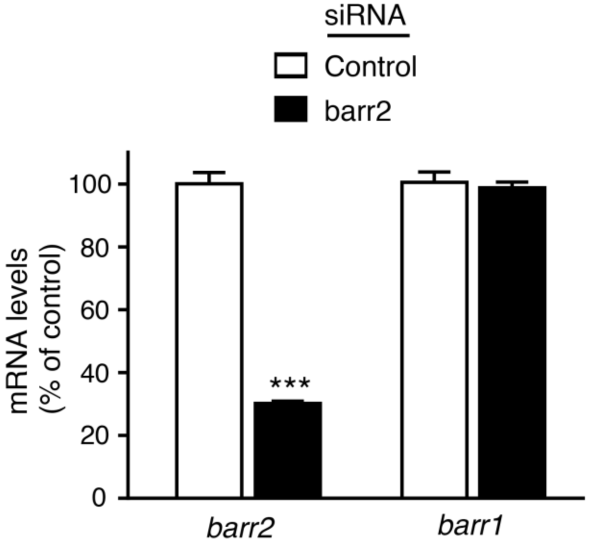

Supplementary Figure 10. Effective knockdown of *barr2* expression in MIN6 cells by *barr2* siRNA. MIN6 cells were electroporated with *barr2* siRNA or scrambled control siRNA. *barr2* and *barr1* mRNA levels were determined ~48 hr later via real-time qRT-PCR. Data were normalized relative to the expression of *β-acti*n. In each individual experiment, *barr2* and *barr1* mRNA levels obtained with cells treated with control siRNA were set equal to 100%. Data are expressed as means ± s.e.m. from three independent experiments. ***$p<0.001$ (Student's t-test).

.

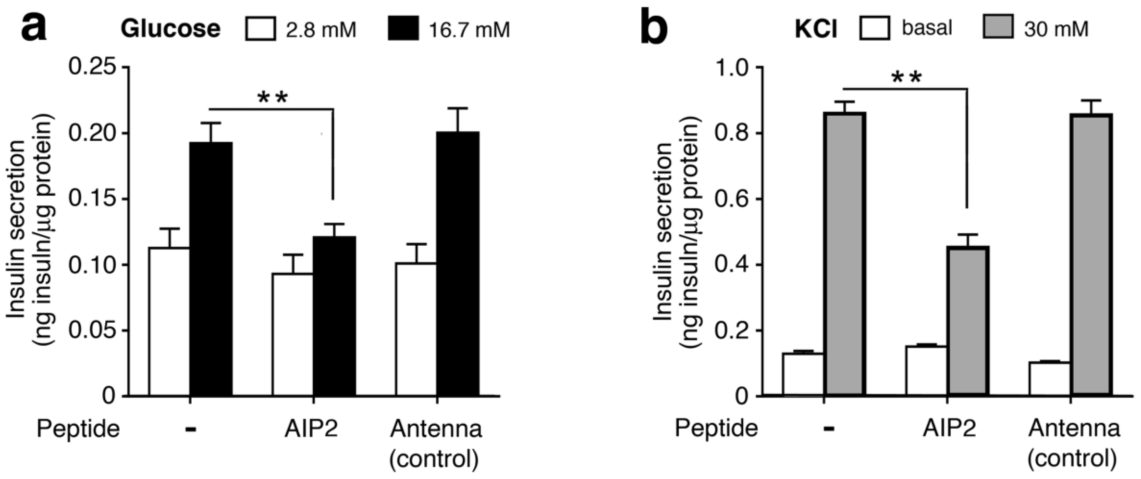

Supplementary Figure 11. A membrane-permeable control peptide (Antenna) has no effect on glucose- or KCl-stimulated insulin secretion in MIN6 cells. (**a**) Glucose (16.7 mM)-stimulated insulin secretion. (**b**) KCl (30 mM)-stimulated insulin secretion. As expected, glucose- and KCl-stimulated insulin secretion were significantly reduced in the presence of the selective CAMKII inhibitor, AIP2 (5 µM). In contrast, stimulated insulin secretion remained unaffected by the 'Antenna' control peptide (antennapedia homeodomain leader peptide; 5 µM) which, like AIP2, can cross the plasma membrane and is part of the AIP2 peptide. Data are given as means ± s.e.m. from three independent experiments carried out in triplicate. **$p<0.01$, as compared to the indicated control group (two-way ANOVA followed by Tukey's post-test).

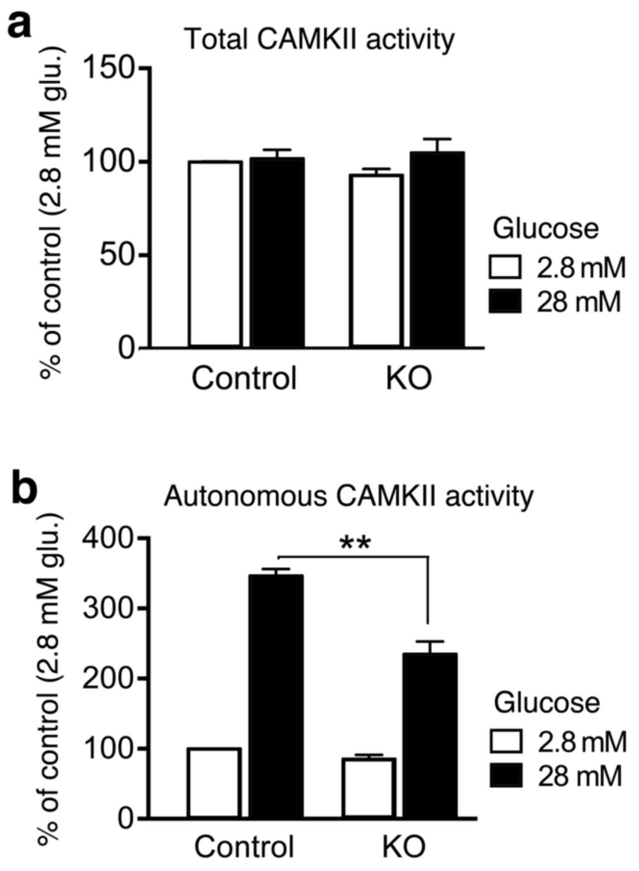

Supplementary Figure 12. Pancreatic islets from β-barr2-KO mice show reduced autonomous CaMKII activity. Control or β-barr2-KO islets were incubated for 2.5 min at 37 °C in the presence of the indicated glucose concentrations (low, 2.8 mM; high, 28 mM). Islet lysates were then used for CaMKII activity assays (see Methods for details). Total CAMKII activity was determined in the presence of Ca^{2+}/calmodulin. Autonomous (Ca^{2+}-independent) CAMKII activity was assessed in the presence of EGTA and the absence of Ca^{2+}/calmodulin. (**a**) Barr2 deficiency has no effect on total CaMKII activity. (**b**) Lack of barr2 causes a significant reduction of autonomous CaMKII activity in the presence of 28 mM glucose. In each individual experiment, the total or autonomous CAMKII activity observed with control islets at 2.8 mM glucose was set equal to 100% (means ± s.e.m. from three independent experiments). Absolute control CAMKII activities at 2.8 mM glucose were (in pmol/min/μg protein): (a), 3.37±0.30; (b), 0.29±0.03; **p<0.01, as compared to the indicated control group (two-way ANOVA followed by Tukey's post-test).

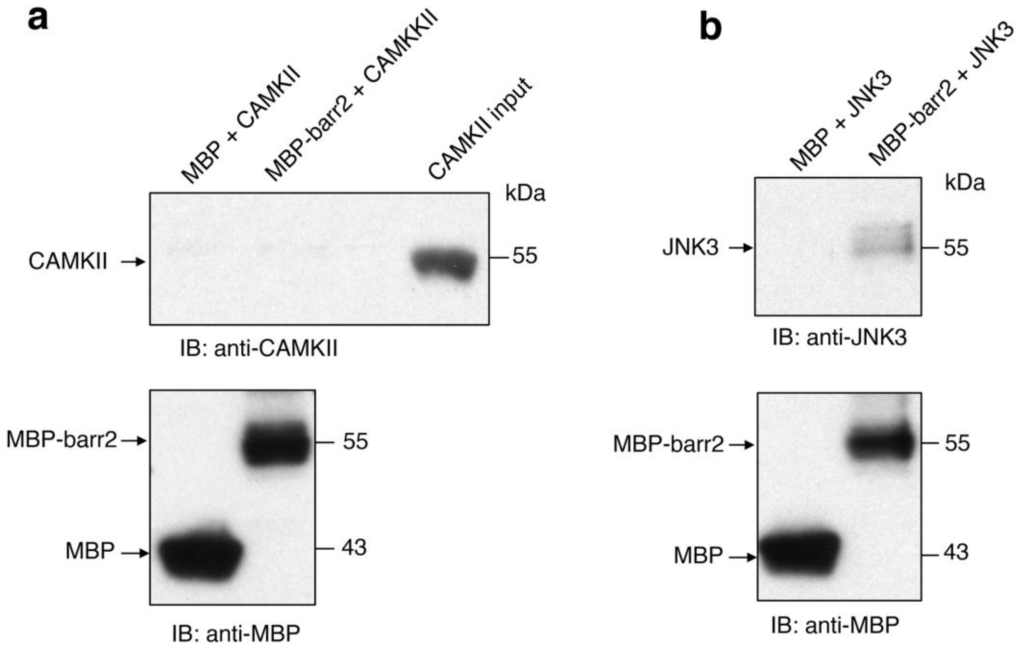

Supplementary Figure 13. Purified CaMKII and barr2 do not interact with each other directly. Purified MBP or MBP-barr2 were bound to amylose beads, followed by the addition of purified CAMKII (CAMKIIδ) (**a**) or purified JNK3 (JNK3α2) (**b**). Bound proteins were eluted with a maltose-containing buffer and subjected to SDS-PAGE and Western blotting. This approach confirmed that JNK3 can bind to barr2 (positive control) (b), but failed to demonstrate a direct interaction of CaMKII with barr2 (MBP-barr2) (a). MBP pull-down assays were carried out as described under Methods. Representative blots from three independent experiments are shown.

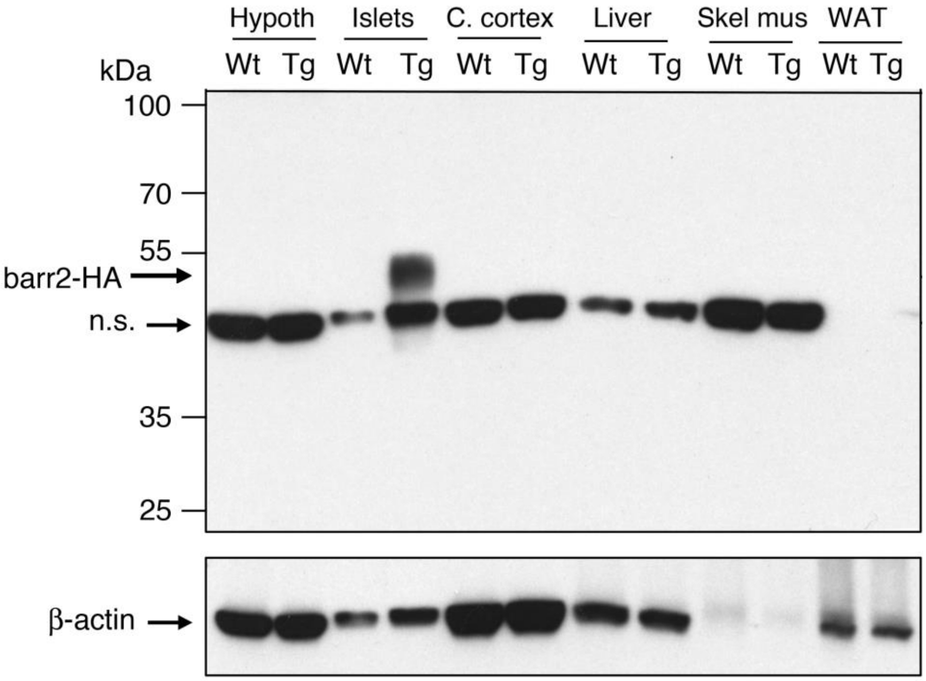

Supplementary Figure 14. Islet (β-cell)-specific overexpression of barr2 in *RIPII-barr2* transgenic mice. Selective overexpression of barr2 in islets of *RIPII-barr2* transgenic mice was verified via Western blotting. Immunoblots were probed with an anti-HA antibody that recognizes an HA epitope tag that was fused to the C-terminus of the *barr2* transgene. Note that the antibody detects only one specific band of the expected size (~45-50 kDa) in islets from the transgenic mice. 'n.s.' denotes a non-specific band seen in nearly all tissues independent of mouse genotype. A representative Western blot is shown.

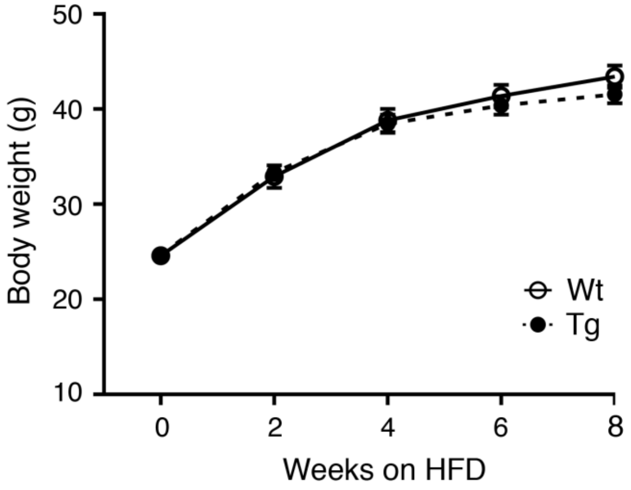

Supplementary Figure 15. Overexpression of barr2 in β-cells has no effect on body weight gain when mice are maintained on a high-fat diet. *RIPII-barr2* transgenic (Tg) mice and wt littermates (males) were maintained on a high-fat diet (HFD) for 8 weeks. Data are given as means ± s.e.m. (8 or 9 per group).

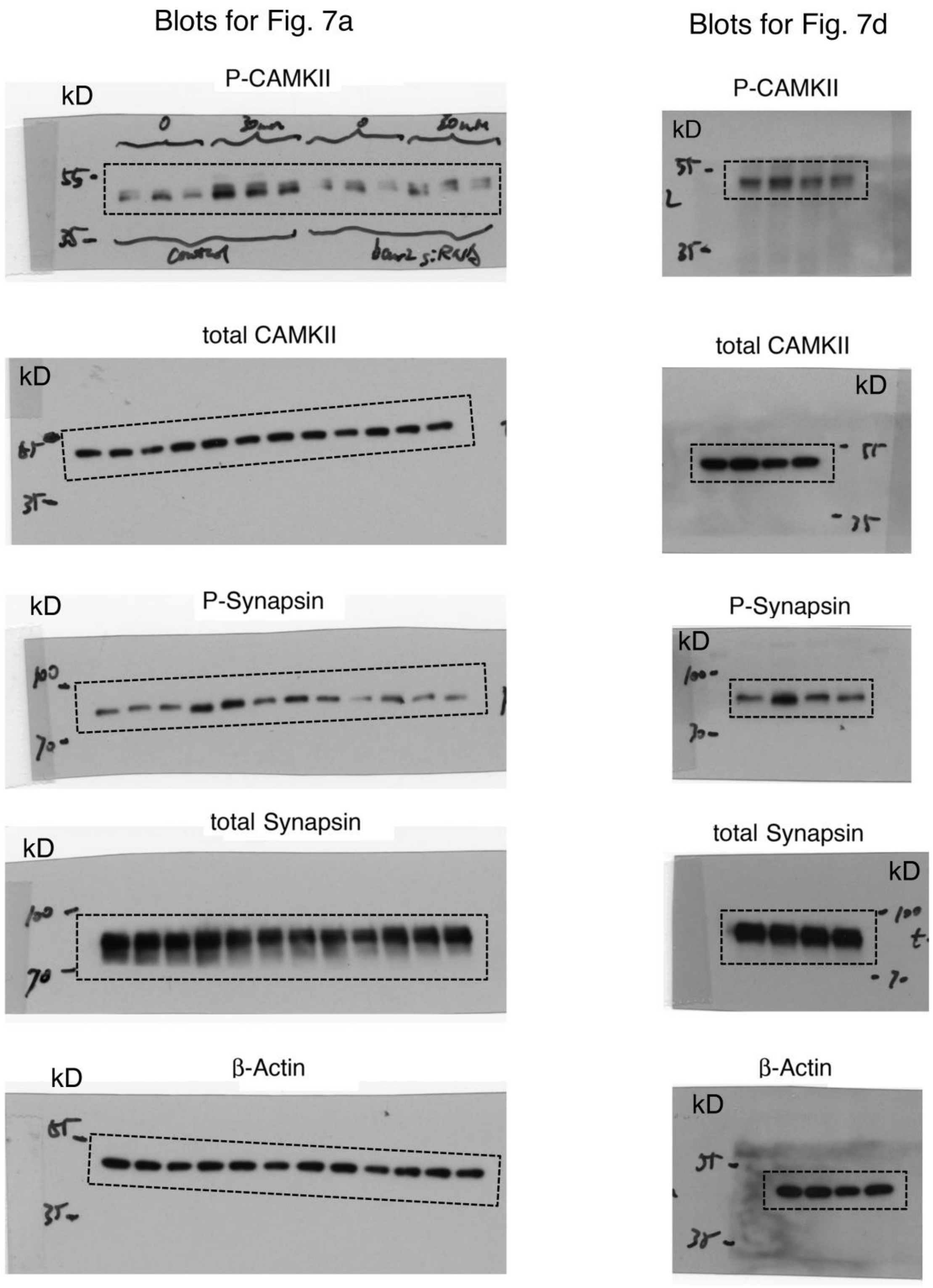

Supplementary Figure 16. Blots correspond to those shown in Figure 7a, d in the main manuscript.

Blots for Fig. 8a

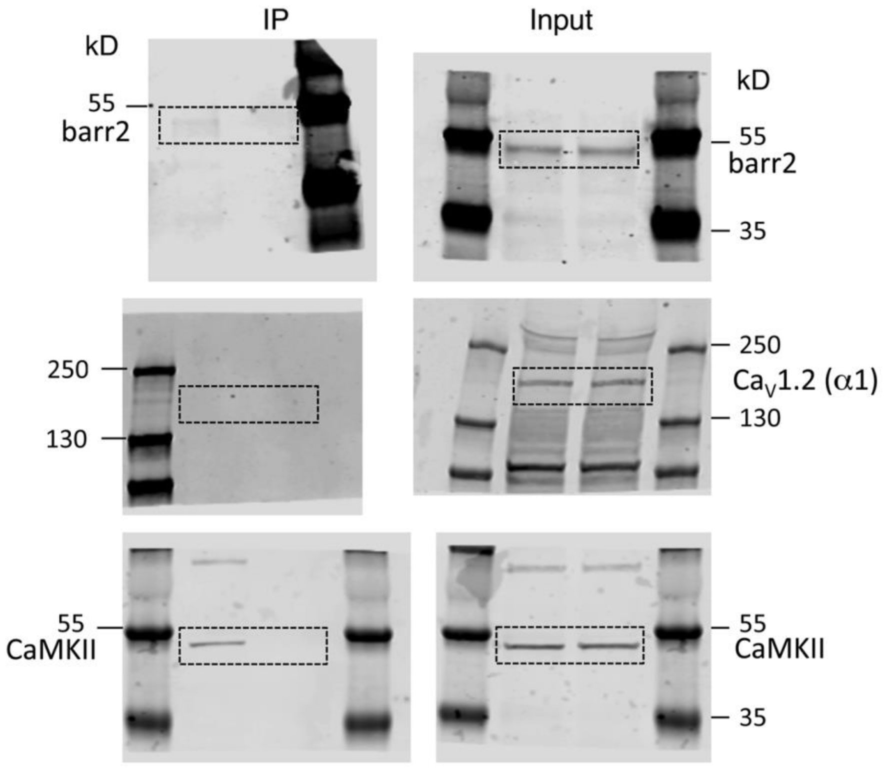

Blots for Fig. 8b

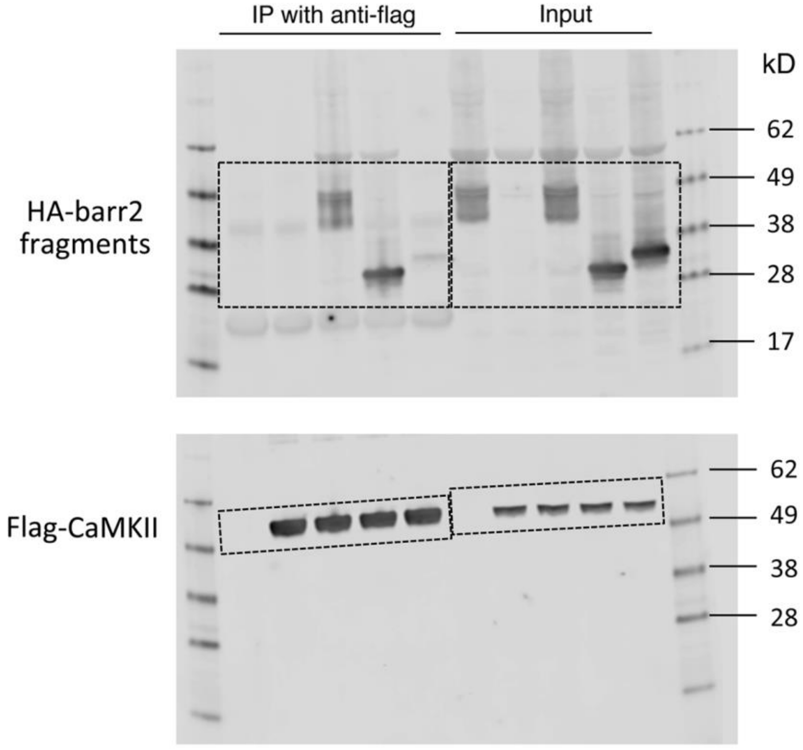

Supplementary Figure 17. Blots correspond to those shown in Figure 8a, b in the main manuscript.

Supplementary Table 1 Blood glucose and plasma insulin levels of β-barr2-KO and *RIPII-barr2* Tg mice and their control littermates

	Regular Chow		HFD	
	Control	β-barr2-KO	Control	β-barr2-KO
Blood glucose (fed, mg dl^{-1})	139 ± 3	158 ± 7 *	168 ± 12	236 ± 29 *
Blood glucose (fasted, mg dl^{-1})	68 ± 4	73 ± 4	99 ± 7	149 ± 10 **
Plasma insulin (fed, ng ml^{-1})	2.27 ± 0.37	1.92 ± 0.22	12.61 ± 0.82	10.09 ± 1.04
Plasma insulin (fasted, ng ml^{-1})	0.62 ± 0.10	0.58 ± 0.06	2.67 ± 0.15	2.44 ± 0.23
	Regular Chow		HFD	
	Wt	RIP2-barr2-Tg	Wt	RIP2-barr2-Tg
Blood glucose (fed, mg dl^{-1})	150 ± 7	126 ± 4 **	187 ± 7	144 ± 6 **
Blood glucose (fasted, mg dl^{-1})	69 ± 2	62 ± 3	134 ± 13	92 ± 8 *
Plasma insulin (fed, ng ml^{-1})	1.75 ± 0.30	2.04 ± 0.36	6.69 ± 1.99	8.43 ± 1.65
Plasma insulin (fasted, ng ml^{-1})	0.29 ± 0.05	0.29 ± 0.03	1.57 ± 0.37	2.08 ± 0.44

Blood (plasma) was collected from male mice that had free access to food (fed) or had been fasted for 12 hr overnight. Mice were maintained on either regular chow or a high-fat diet (HFD). Data are given as means ± s.e.m. (7-10 mice per group; mouse age: regular chow mice, ~12 weeks; HFD mice, ~20-weeks).). *p<0.05, **, p<0.01, as compared to the corresponding control value (Student's t-test).

Supplementary Table 2 Summary of antibodies used for immunoblotting (IB), immunoprecipitation (IP), and immunohistochemical (IHC) studies

Antibody target	Source of antibody	Catalog #	Dilution	Usage
Phospho-CAMKII	Cell Signaling Technology	3361	1:1,000	IB
CAMKII (pan)	Cell Signaling Technology	3362	1:1,000	IB
CAMKIIδ	Santa Cruz	sc-5392	1:100	IP
β-arrestin-2	Cell Signaling Technology	3857	1:1,000	IB
β-arrestin-1/2	Cell Signaling Technology	4647	1:1,000	IB
$Ca_V1.2$ (α1 subunit)	Amy Lee lab[1]		1:1,000	IB
Phospho-synapsin	Cell Signaling Technology	2311	1:1,000	IB
Synapsin	Cell Signaling Technology	5297	1:1,000	IB
JNK3	Cell Signaling Technology	2305	1:1,000	IB
MBP tag	Cell Signaling Technology	2396	1:1,000	IB
β-actin	Cell Signaling Technology	3700	1:2,000	IB
HA tag	Cell Signaling Technology	3724	1:1,000	IB
c-myc tag	Santa Cruz	sc-40	1:1,000	IB
Flag tag	Sigma-Aldrich	F1804	1:3,000	IB
Flag tag	GenScript Corp.	A00187-200	1:100	IP
Anti-rabbit IgG, HRP-linked secondary antibody	Cell Signaling Technology	7074	1:3,000	IB
Anti-mouse IgG, HRP-linked secondary antibody	Cell Signaling Technology	7076	1:3,000	IB
Insulin (guinea pig) polyclonal antibody)	Thermo Fisher Scientific	PA1-26938	1:100	IHC
Glucagon (rabbit polyclonal antibody)	Thermo Fisher Scientific	RB-1422-A1	1:100	IHC
Alexa Fluor 555 goat anti-guinea pig	Invitrogen	A21435	1:500	IHC
Alexa Fluor 488 goat anti-rabbit	Invitrogen	A11034	1:500	IHC

Supplementary Table 3 Summary of primers used for qRT-PCR experiments

**Mouse gene**	**Primer sequence**	**Amplicon (bp)**
β-actin	QuantiTect Primer (Qiagen)<br>Cat. No.: QT01136772	77
GAPDH	Forward: 5’ ACAGTCCATGCCATCACTGCC<br>Reverse: 5’ GCCTGCTTCACCACCTTCTTG	266
Barr2	Forward: 5’ GTCTTCAAGAAGTCGAGCCCT<br>Reverse: 5’ CACGAACACTTTCCGGTCCT	144
Barr1	QuantiTect Primer (Qiagen)<br>Cat. No.: QT00152880	77
Glut2	Forward: 5’ CATTCTTTGGTGGGTGGC<br>Reverse: 5’ CCTGAGTGTGTTTGGAGCG	221
Ins2<br>*(Preproinsulin 2)*	Forward: 5’ CTGGCCCTGCTCTTCCTCTGG<br>Reverse: 5’ CTGAAGGTCACCTGCTCCCGG	204
Irs2	Forward: 5’ CTGCGTCCTCTCCCAAAGTG<br>Reverse: 5’ GGGGTCATGGGCATGTAGC	124
Pcx<br>*(Pyruvate*<br>*carboxylase)*	Forward: 5’ CTGAAGTTCCAAACAGTTCGAGG<br>Reverse: 5’ CGCACGAAACACTCGGATG	162
Pdx1	Forward: 5’ CCCCAGTTTACAAGCTCGCT<br>Reverse: 5’ CTCGGTTCCATTCGGGAAAGG	177
Stx1a<br>*(Qc-SNARE)*	QuantiTect Primer (Qiagen)<br>Cat. No.: QT00101514	109
Snap25<br>*(Qa-Qb SNARE)*	QuantiTect Primer (Qiagen)<br>Cat. No.: QT01658391	147
Vamp2<br>*(R-SNARE)*	QuantiTect Primer (Qiagen)<br>Cat. No.: QT00256942	92
Sur1<br>*(Abcc8)*	QuantiTect Primer (Qiagen)<br>Cat. No.: QT01042300	77
Ca$_V$*1.2* (□)	QuantiTect Primer (QIAGEN)	115

	Cat. No.: QT00150752	
Ca_V1.3 (□)	QuantiTect Primer (QIAGEN)<br>Cat. No.: QT00112238	96
Epac1	Forward: 5’ TCTTACCAGCTAGTGTTCGAGC<br>Reverse: 5’ AATGCCGATATAGTCGCAGATG	223
Epac2-A	Forward: 5’ CAAGGAGAATGTGAGTAGCCAC<br>Reverse: 5’ TCGCTGTGCTTATGTTTTACCT	75
Epac2-B	Forward: 5’ ACCTCTCATTGAACCCCACG<br>Reverse: 5’ GAAGGGACCTTGGTAATGGTG	60
**Human gene**	**Primer sequence**	**Amplicon (bp)**
BARR2 (ARRB2)	QuantiTect Primer (Qiagen)<br>Cat. No.: QT00058051	89
BARR1 (ARRB1)	QuantiTect Primer (Qiagen)<br>Cat. No.: QT00071197	123
GAPDH	QuantiTect Primer (Qiagen)<br>Cat. No.: QT00079247	95
β-ACTIN (ACTB)	Forward: 5’ GATCATTGCTCCTCCTGAGC<br>Reverse: 5’ ACTCCTGCTTGCTGATCCAC	101

Supplementary Reference

1. Tippens, A.L. *et al.* Ultrastructural evidence for pre- and postsynaptic localization of $Ca_v1.2$ L-type Ca^{2+} channels in the rat hippocampus. *J. Comp. Neurol.* 506, 569-583 (2008).
